# Association of the *ACTN3* R577X (rs1815739) polymorphism with elite power sports: A meta-analysis

**DOI:** 10.1371/journal.pone.0217390

**Published:** 2019-05-30

**Authors:** Phuntila Tharabenjasin, Noel Pabalan, Hamdi Jarjanazi

**Affiliations:** 1 Chulabhorn International College of Medicine, Thammasat University, PathumThani, Thailand; 2 Environmental Monitoring and Reporting Branch, Ontario Ministry of the Environment, Conservation and Parks, Toronto, Ontario, Canada; University of Umeå, SWEDEN

## Abstract

**Objective:**

The special status accorded to elite athletes stems from their uncommon genetic potential to excel in world-class power sports (PS). Genetic polymorphisms have been reported to influence elite PS status. Reports of associations between the *α-actinin-3* gene (*ACTN3*) R577X polymorphism and PS have been inconsistent. In light of new published studies, we perform a meta-analysis to further explore the roles of this polymorphism in PS performance among elite athletes.

**Methods:**

Multi-database literature search yielded 44 studies from 38 articles. Pooled odds ratios (ORs) and 95% confidence intervals (CIs) were used in estimating associations (significance threshold was set at P^a^ ≤ 0.05) using the allele-genotype model (R and X alleles, RX genotype). Outlier analysis was used to examine its impact on association and heterogeneity outcomes. Subgroup analysis was race (Western and Asian) and gender (male/female)-based. Interaction tests were applied to differential outcomes between the subgroups, P-values of which were Bonferroni corrected (P_interaction BC_). Tests for sensitivity and publication bias were performed.

**Results:**

Significant overall R allele effects (OR 1.21, 95% CI 1.07–1.37, P^a^ = 0.002) were confirmed in the Western subgroup (OR 1.11, 95% CI 1.01–1.22, P^a^ = 0.02) and with outlier treatment (ORs 1.12–1.20, 95% CIs 1.02–1.30, P^a^ < 10^−5^–0.01). This treatment resulted in acquired significance of the RX effect in Asian athletes (OR 1.91, 95% CI 1.25–2.92, P^a^ = 0.003). Gender analysis dichotomized the RX genotype and R allele effects as significantly higher in male (OR 1.14, 95% CI 1.02–1.28, P^a^ = 0.02) and female (OR 1.58, 95% CI 1.21–2.06, P^a^ = 0.0009) athletes, respectively, when compared with controls. Significant R female association was improved with a test of interaction (P_interaction BC_ = 0.03). The overall, Asian and female outcomes were robust. The R allele results were more robust than the RX genotype outcomes. No evidence of publication bias was found.

**Conclusions:**

In this meta-analysis, we present clear associations between the R allele/RX genotype in the *ACTN3* polymorphism and elite power athlete status. Significant effects of the R allele (overall analysis, Western and female subgroups) and RX genotype (Asians and males) were for the most part, results of outlier treatment. Interaction analysis improved the female outcome. These robust findings were free of publication bias.

## Introduction

Exceptional performance during national/international-level competitions defines elite athletes [1]. Among elite power sports (PS) athletes, performance varies across countries, races and gender. This variation stems from genetic and environmental factors. It has been reported that genes play a substantial role in muscle function, specifically those involving the PS phenotype [2,3]. Genetic variants (i.e. polymorphisms) are vital in understanding the potential influence of genes on PS [4] but published outcomes have been variable [5,6]. Nevertheless, lines of evidence have catapulted the *ACTN3* (*α-actinin-3*) polymorphism into prominence regarding its association with PS performance [7,8].

*ACTN3* is the gene that encodes for *α-actinin-3*, which is a sarcomeric protein expressed mainly in type II muscle fibers [9]. Type-II muscle fibers are involved in generating explosive and powerful muscle contractions. A common polymorphism in this gene is *ACTN3* R577X (rs1815739), where a cytosine-to-thymine base substitution transforms the arginine base (R) to a premature stop codon (X). The X allele is a loss-of-function variant wherein homozygosity results in a complete lack of expression of *α-actinin-3*, the deficiency of which occurs in ~20% of the world’s population [8]. Given functional de-emphasis on the X allele, greater attention has been paid to the relationship of the R allele with PS performance [10] where athletes with the RR genotype are associated with high muscle strength [11] and power [12]. In line with these findings, we examine the hypothesis that the RR genotype is more common among sprint/power athletes compared with their controls [4,13].

The popularity of *ACTN3* in sports performance is attested by its coverage in several reviews [7,8,14,15]. Current knowledge regarding associations of the *ACTN3* R577X polymorphism with sports performance involves investigation of its polygenic nature and interactions [16–20]. Thus, *ACTN3* literature has focused on association analyses between several genes related to sports performance [21,22]. Genome-wide association studies (GWAS) involving *ACTN3* is an emerging possibility in the genomics of sports science. More commonly in *ACTN3* R577X research, however, is the study of the R and X alleles as well as RX genotype frequencies between athlete and control; between sport types, mainly power and endurance and comparisons between populations. Studies have found that the R allele is more frequent in power athletes than controls [23] and in power more than endurance [24]. However, other studies on PS performance showed no associations, rendering inconsistency in the outcomes [22,25,26].

Meta-analysis is a potent methodology that synthesizes the results of independent studies thus increasing statistical power and resolution. Our rationale for doing this meta-analysis rests on the following: (i) the synthetic approach gives a simultaneous overview and detailed profiles of the role of *ACTN3* R577X in PS performance which is facilitated by an arsenal of meta-analysis tools that can be used; (ii) primary study outcomes on this topic have been inconsistent with associations found in some reports [4,26,27] and none in others [28–30] and (iii) new studies have since been published. In this study, we aim to determine the role of *ACTN3* R577X in the PS phenotype among elite power athletes.

## Materials and methods

### Selection of studies

**Fig 1:**
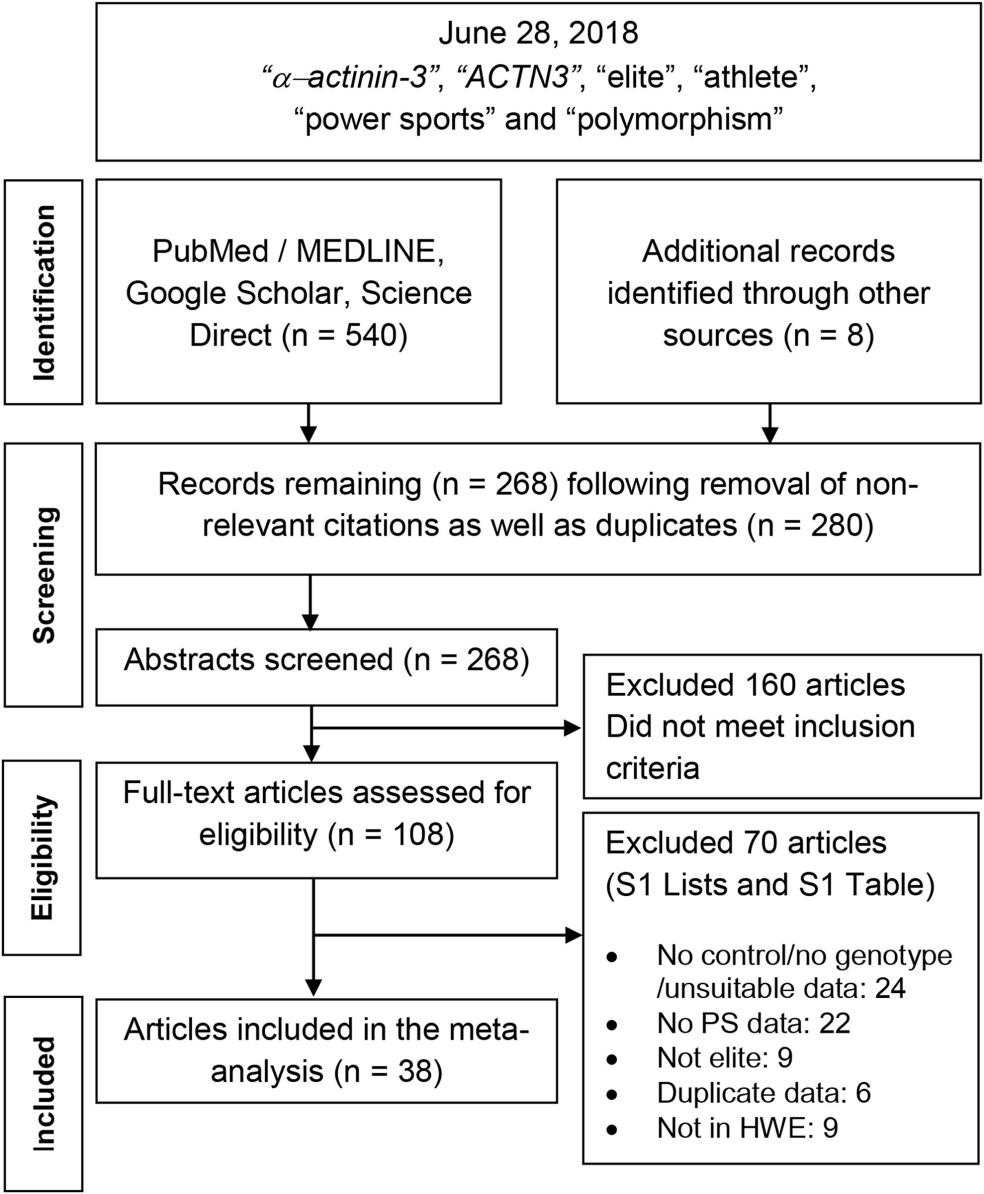
Summary flow chart of literature search. *ACTN3*: *alpha-actinin-3*; HWE: Hardy-Weinberg Equilibrium; PS: power sports.

We searched MEDLINE using PubMed, Science Direct and Google Scholar for association studies as of June 28, 2018. The terms used were *“α-actinin-3*”, “*ACTN3*”, “elite”, “athlete”, “power sports” and “polymorphism” as medical subject headings and text, restricted to the English language. References cited in the retrieved articles were also screened manually to identify additional eligible studies. The exclusion criteria were; (i) review; (ii) subjects were non-human; (iii) non-English; (iv) studies did not involve PS performance (e.g. *ACTN3* polymorphism effects in disease conditions or cases were non-athletes) and (v) not *ACTN3* by title/abstract. Other exclusion criteria are listed in Fig 1 and S1 List. The inclusion criteria were: (i) case–control study design evaluating the association between *ACTN3* R577X polymorphism and elite power athletes; (ii) sufficient genotype frequency data to calculate the odds ratios (ORs) and 95% confidence intervals (CIs) and (iii) controls in studies comply with the Hardy-Weinberg Equilibrium (HWE) which was assessed using the online application (https://ihg.gsf.de/cgi-bin/hw/hwa1.pl). S1 Table details information about the excluded articles based non-compliance with HWE.

### Data extraction

Two investigators (PT and NP) independently extracted data and arrived at a consensus. Authors were contacted in order to obtain more information on incomplete data. The following information was extracted from each article: first author’s name, published year, country of origin, race and gender of participants, those that addressed gene-gene, gene-environment interactions, those that used haplotype analysis, matching and sample source. Reference lists of screened full-text articles were scanned for additional studies. Where articles reported multiple genotypes under PS levels, we chose those that referred to “elite” or “international” or otherwise contextualized the status of elite athletes according to Lorenz et al [31]. Departures of genotypic frequencies from the HWE in control subjects were determined with the χ^2^ test.

### Quality assessment of the studies

The Clark-Baudouin (CB) scale was used to evaluate methodological quality of each included study [32]. Suitability of this scale is based on criteria that include P-values, addressing statistical power issues, corrections for multiple testing, sample size comparisons between cases and controls, use of primers and detailing of genotyping methods. CB scores range from zero (worst) to 10 (best) with low and high quality designated as < 5 and ≥ 5, respectively.

### Data distribution and publication bias

Data distribution was assessed with the Lilliefors corrected-Kolmogorov-Smirnov (KS) test [33] using SPSS 20.0 (SPSS Inc., IBM Corp. Chicago, IL, USA). Descriptive statistics of normally distributed data were expressed as mean ± standard deviation (SD), otherwise, we used median and interquartile range (IQR). We assessed publication bias using WINPEPI [34]. Study-specific ORs were used as operating data for publication bias tests, the choice of which depended on their distribution. Inferential protocol warranted the Egger’s test [35] for normally distributed data, otherwise, the Begg-Mazumdar test was used [36].

### Data synthesis

We estimated associations (OR) and 95% CI among elite athletes for each study. Where genotype values were zero, we applied the Laplace correction by adding a pseudo-count of one to all values of the data set [37] before constructing the forest plots. Because frequency of the variant X allele was non-uniform, we compared the following: (i) X allele with RX/RR genotype; (ii) R allele with RX/XX genotype and (iii) RX genotype with homozygous RR and XX genotypes (heterozygote comparison). This modeling approach facilitates comparisons and interpretations of findings with *ACTN3* literature. Raw data for genotype frequencies, without adjustment, were used to calculate study-specific ORs. To assess the strength of evidence, we used the following indicators: (i) magnitudes of effects are stronger or weaker when the values are closer to or farther from the non-associative OR of 1.0, respectively; (ii) precision of effects was assessed with the confidence interval difference (CID = upper CI—lower CI). High and low CID values indicate low and high precision, respectively and (iii) P-values close to 0.05 and 0.0001 indicate moderate and high significance, respectively. Two-sided P-values of ≤ 0.05 were considered significant except in estimations of heterogeneity where the threshold was set at ≤ 0.10 on account of the low power of the χ^2^-based Q test [38]. Heterogeneity between studies was estimated with the χ^2^-based Q test [39], explored with subgroup analysis [39] and quantified with the I^2^ statistic which measures degree of inconsistency among studies [40]. Sources of heterogeneity were detected using the Galbraith plot [41]. Outlier treatment involves omitting the sources of heterogeneity (outliers) followed by reanalysis [42]. Less variability in the study characteristics [43] of the component studies indicate reduced heterogeneity [44]. This warranted use of the fixed-effects model [45], otherwise, we opted for the random-effects model [46]. Sensitivity analysis, which involves omitting one study at a time and recalculating the pooled OR, was used to test for robustness of the summary effects. Differential outcomes between the races and genders warranted the test of interaction [47]. The concept of this test is contextualize the pooled effect of one subgroup (already established by the test of overall effect) by comparing it with its counterpart subgroup (its association independently obtained). In this meta-analysis, the test of interaction involves comparing the pooled ORs and their 95% CIs with their counterparts in the race and gender subgroups (e.g. Western versus Asian, Western versus African, female versus male). A significant Bonferroni corrected P-value of interaction (P_BC_ < 0.05) from the z-test means improved power of association. Data were analyzed using Review Manager 5.3 (Cochrane Collaboration, Oxford, England), SIGMASTAT 2.03, SIGMAPLOT 11.0 (Systat Software, San Jose, CA).

## Results

### Search results and study features

Fig 1outlines the study selection process in a flowchart following PRISMA (Preferred Reporting Items for Systematic Reviews and Meta-Analyses) guidelines. A total of 548 citations during the initial search were subjected to a series of omissions. S2 Table shows the 38 articles which includes a published thesis [48]. Of the 38, 16 (*) were not included (new) in the 4 previous meta-analyses [10,13,49,50]. Year range of the articles was 2003–2018.

CB score distributions were non-normal (KS: P ≤ 0.002), where median for the 38 studies was 7.0 (IQR 6.0–7.0) indicating high methodological quality of the articles. Confined to the 16 new articles (42.1%), median CB was higher at 8.0 (IQR 7.0–8.0). However, the difference between CB scores in the 38 and 16 studies was marginal (Mann-Whitney U test: P = 0.06). Proportion of articles using blood (65.8%) was higher than those that used saliva or buccal (57.9%) as sources for genotyping. Of note, 9 articles (23.7%) used both sources. Multiple datasets from 5 publications placed the total number of studies to 44 (S2 Table). Of the 44 studies, 31 were Western (70.4%), 9 Asian (20.5%) and 4 African (9.0%) subjects.

S3 Table shows quantitative features of the 44 studies which include sample sizes, number of cases and controls, genotype frequencies, including the minor allele and P-values for HWE. A detailed description of this meta-analysis is summarized in the checklists for PRISMA (S4 Table) and for genetic association studies (S5 Table).

### Meta-analysis outcomes

**Fig 2:**
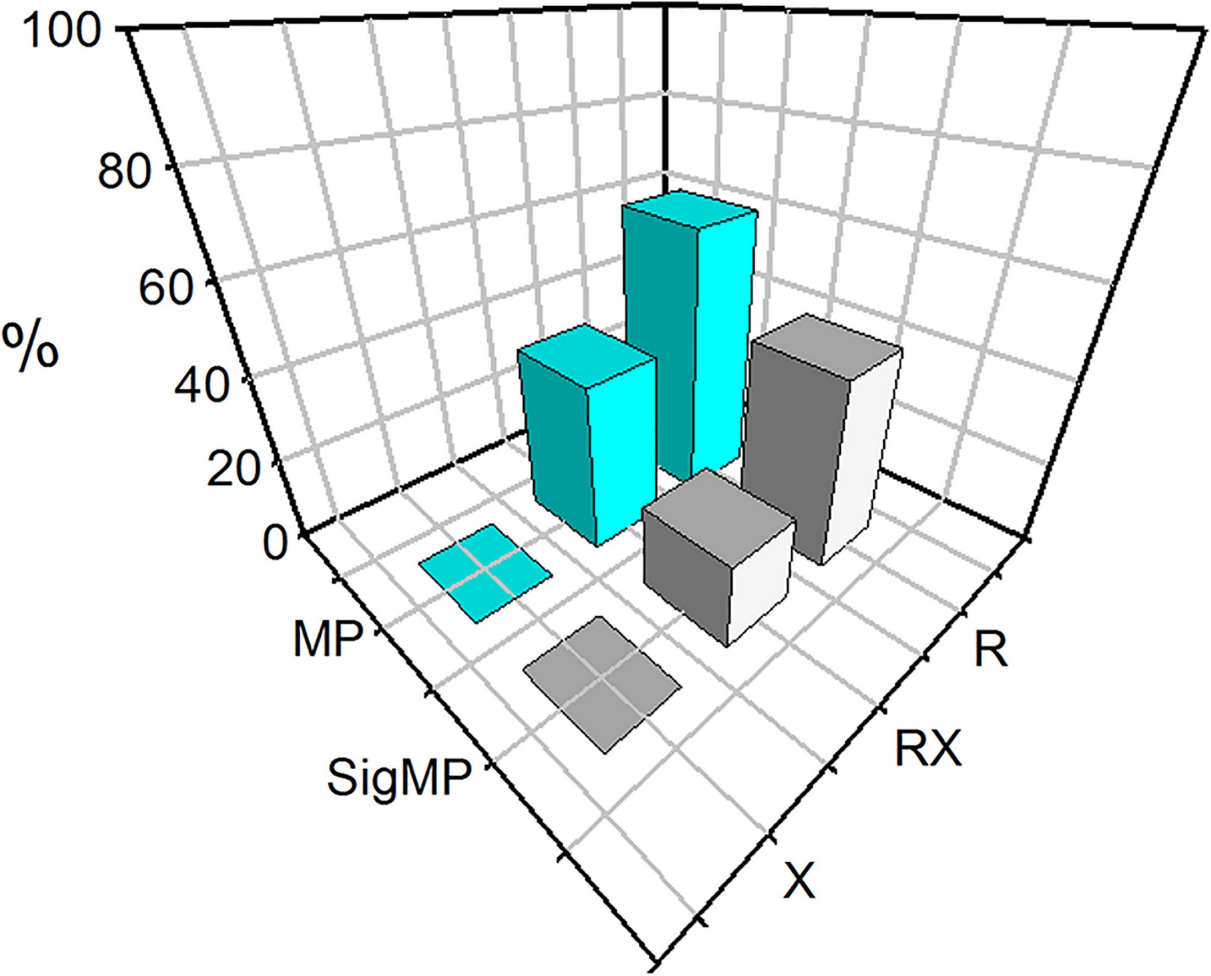
3D plot of *ACTN3* polymorphism effects in power sports. R: wild-type; X: variant-type; RX: heterozygous; MP: more in power; SigMP: significantly more in power.

Here, we emphasize on reporting pooled effects found more in power (MP) rather than in controls (MC). There are two reasons for this emphasis, first, is that power-leaning (MP) effects allow interpretation of results that quantify the magnitude of association. The second reason relates to de-emphasizing the X allele effects which Table 1 and Fig 2 show as non-associative (null/MC) in all comparisons. We thus focus on the R allele and RX genotype where significant associative outcomes (P < 0.05) were observed (Fig 2).

**Table 1:** Overall analysis of R allele in *ACTN3* R577X polymorphism with power sports.

	Test of association					Test of heterogeneity				Test of association					Test of heterogeneity				Effect of outlier treatment	
	n	OR	95% CI	CID	P^a^	Sports Performance	P^b^	I^2^ (%)	AM	n	OR	95% CI	CID	P^a^	Sports Performance	P^b^	I^2^ (%)	AM	Signi-ficance	Hetero geneity
	PRO									PSO										
Overall power																				
R	44	**1.21**	**1.07–1.37**	0.30	**0.002**	MP	10^−5^	61	RE	39	**1.20**	**1.12–1.30**	0.18	**10**^**−5**^	MP	0.10	23	F	ES	RH
X	44	0.71	0.62–0.82	0.20	10^−5^	MC	0.01	35	RE	42	0.71	0.64–0.79	0.15	10^−5^	MC	0.11	22	F	---	---
RX	44	0.90	0.80–1.01	0.21	0.08	MC	10^−5^	62	RE	38	0.96	0.89–1.04	0.15	0.30	MC	0.10	24	F	---	---
Western																				
R	31	**1.11**	**1.01–1.22**	0.21	**0.02**	MP	10^−4^	60	RE	28	**1.12**	**1.02–1.23**	0.21	**0.01**	MP	0.22	16	F	RS	RH
X	31	0.78	0.65–0.92	0.27	0.004	MC	0.02	39	RE	30	0.81	0.71–0.92	0.21	0.001	MC	0.13	23	F	---	---
RX	31	0.93	0.80–1.07	0.27	0.31	MC	10^−5^	63	RE	28	1.03	0.95–1.13	0.18	0.45	Null	0.12	25	F	RNS	RH
Asian																				
R	9	0.83	0.48–1.43	0.95	0.49	MC	10^−5^	92	RE	7	1.11	0.65–1.89	1.29	0.69	MP	10^−5^	88	RE	RNS	NC
X	9	0.63	0.47–0.83	0.36	0.001	MC	0.01	60	RE	7	0.72	0.56–0.92	0.36	0.008	MC	0.09	45	RE	---	---
RX	9	1.43	0.86–2.39	1.53	0.17	MP	10^−5^	95	RE	7	**1.91**	**1.25–2.92**	1.67	**0.003**	MP	10^−5^	91	RE	GS	NC
African																				
R	4	1.03	0.76–1.40	0.64	0.83	Null	0.52	0	F	---	---	-------	---	---	---	---	---	---	---	---
X	4	0.81	0.34–1.95	1.61	0.64	MC	0.80	0	F	---	---	-------	---	---	---	---	---	---	---	---
RX	4	0.99	0.73–1.36	0.63	0.96	Null	0.48	0	F	---	---	-------	---	---	---	---	---	---	---	---

R: common allele; X: variant allele; RX: heterozygous genotype; n: number of studies; OR: odds ratio; CI: confidence interval; CID: confidence interval difference; P^a^: P-value for association; P^b^: P-value for heterogeneity; I^2^: measure of inconsistency expressed in %; AM: analysis model; PRO: pre-outlier; PSO: post-outlier; F: fixed-effects; RE: random-effects; MP: more in power; MC: more in control; Null (ORs 0.97–1.03); values in bold indicate significant effects on sports performance only (OR > 1.00); the significance and heterogeneity columns were filled when one or both OR values in PRO and/or PSO was MP, otherwise the columns were dashed; ES: elevated significance; RS: retained significance; RNS: retained non-significance; GS: gain in significance; RH: reduced heterogeneity; NC: no change

### Overall and race subgroup

Table 1 shows significant overall associations of the R allele in pre-outlier (PRO) as higher in MP than in MC (OR 1.21, 95% CI 1.07–1.37, P^a^ = 0.002), confirmed in the post-outlier (PSO) analysis (OR 1.20, 95% CI 1.12–1.30, P^a^ < 10^−5^) and in Westerns (ORs 1.11–1.12, 95% CIs 1.01–1.23, P^a^ = 0.01–0.02). Seven (33.3%) of the 21 comparisons in Table 1 were MP (OR > 1.00), 5 (71.4%) of which were significant (P^a^ < 0.05). Of the 5, pooled effects in Asians were the only subgroup to gain significance (GS) and its heterogeneity unaffected by outlier treatment. Initial homogeneity (I^2^ = 0%) of the African non-associative (null/MC) effects (OR 0.81–1.03, 95% CI 0.34–1.95, P^a^ = 0.64–0.96) from 4 studies precluded outlier treatment.

### Gender analysis

Table 2 summarizes a total of 11 comparisons, of which 5 (45.5%) show pooled ORs that were higher in MP than in MC. Two of the 5 were significant (P^a^ < 0.05), both of which were PSO, underpinning the impact of outlier treatment. Two features mark our core findings in gender analysis regarding associations: (i) the RX genotype in males (OR 1.14, 95% CI 1.02–1.28, P^a^ = 0.02) and (ii) the R allele in females (OR 1.58, 95% CI 1.21–2.06, P^a^ = 0.0009), but not in males (ORs 1.01–1.06, 95% CI 0.87–1.20, P = 0.31–0.87).

**Table 2:** Gender analysis in *ACTN3* R577X polymorphism with power sports.

	Test of association					Test of heterogeneity				Test of association					Test of heterogeneity				Effect of outlier treatment	
	n	OR	95% CI	CID	P^a^	Sports Performance	P^b^	I^2^ (%)	AM	n	OR	95% CI	CID	P^a^	Sports Performance	P^b^	I^2^ (%)	AM	Signi-ficance	Hetero-geneity
	PRO									PSO										
Male																				
R	20	1.01	0.87–1.18	0.31	0.87	Null	0.08	33	RE	19	1.06	0.94–1.20	0.26	0.31	MP	0.47	0	F	RNS	EH
X	20	0.75	0.61–0.93	0.32	0.008	MC	0.05	38	RE	19	0.79	0.67–0.94	0.27	0.008	MC	0.35	8	F	---	---
RX	20	1.04	0.87–1.25	0.38	0.66	MP	0.0006	58	RE	19	**1.14**	**1.02–1.28**	0.26	**0.02**	MP	0.45	0	F	GS	EH
Female																				
R	10	1.39	0.93–2.08	1.15	0.11	MP	0.003	63	RE	8	**1.58**	**1.21–2.06**	0.85	**0.0009**	MP	0.11	40	F	GS	RH
X	10	0.98	0.66–1.46	0.80	0.92	Null	0.08	42	RE	9	1.02	0.78–1.31	0.53	0.91	Null	0.10	40	F	RNS	RH
RX	10	0.94	0.77–1.15	0.38	0.54	MC	0.21	25	F	---	---	-------	-----	---	---	---	---	---	---	---

R: common allele; X: variant allele; RX: heterozygous genotype; n: number of studies; OR: odds ratio; CI: confidence interval; CID: confidence interval difference; P^a^: P-value for association; P^b^: P-value for heterogeneity; I^2^: measure of inconsistency expressed in %; AM: analysis model; PRO: pre-outlier; PSO: post-outlier; F: fixed-effects; RE: random-effects; MP: more in power; MC: more in control; Null (ORs 0.97–1.03); values and indicator (SP) in bold indicate significant associations indicating effects on SP only (OR > 1.00); the significance and heterogeneity columns were filled when one or both OR values in PRO and/or PSO was MP, otherwise the columns were dashed; GS: gain in significance; RNS: retained non-significance; EH: eliminated heterogeneity; RH: reduced heterogeneity

### Mechanism of outlier treatment

**Fig 3:**
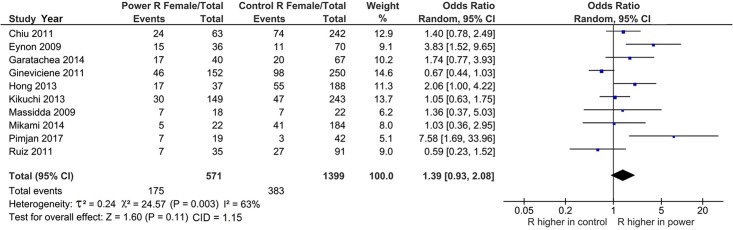
Forest plot of the R allele female effects in the pre-outlier analysis. R: wild-type; CI: confidence interval; P: P-value; χ^2^: chi-square; CID: confidence interval difference.

**Fig 4:**
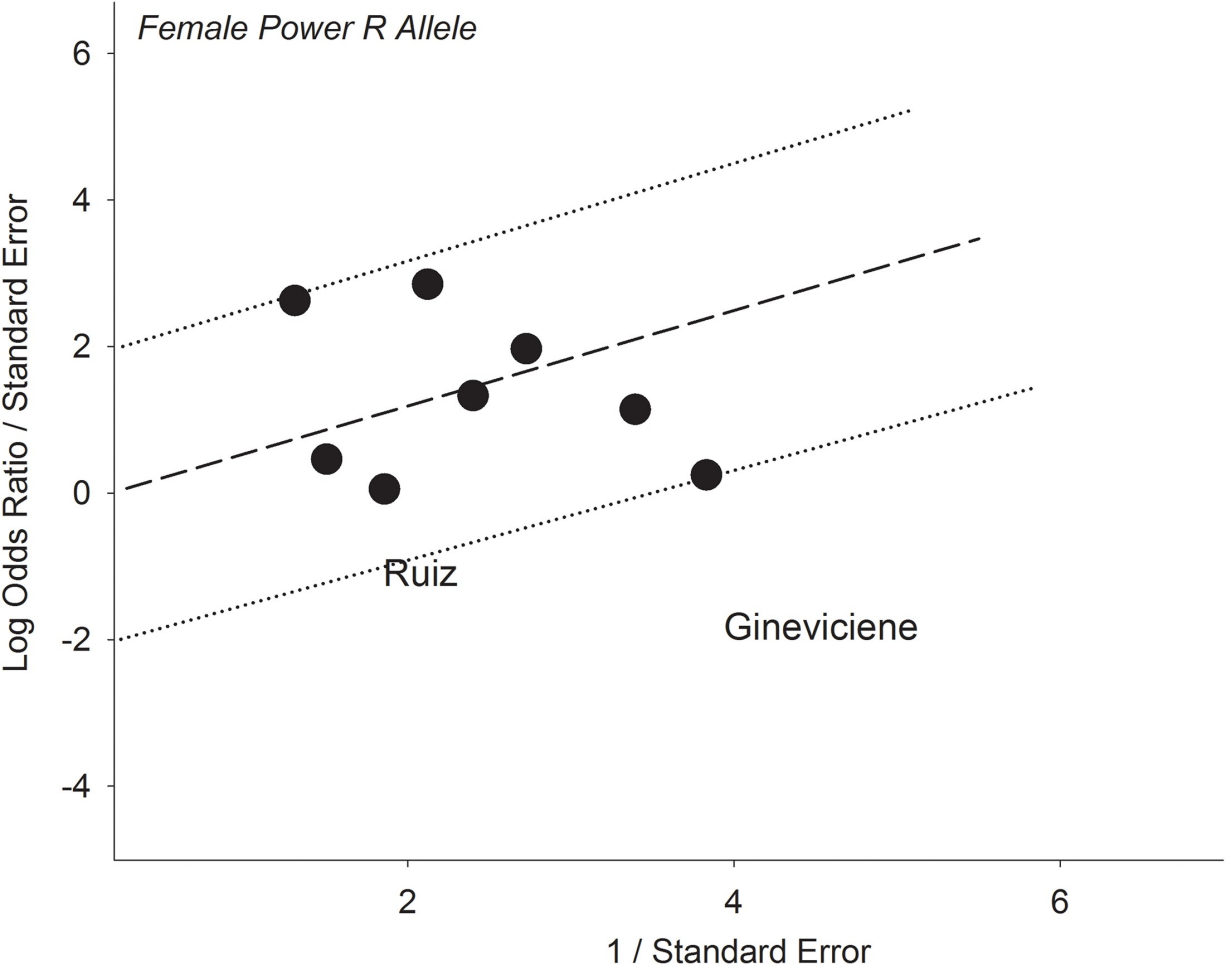
Galbraith plot of the female subgroup in the R allele showing the outlying studies found below the -2 confidence limit. R: wild-type.

**Fig 5:**
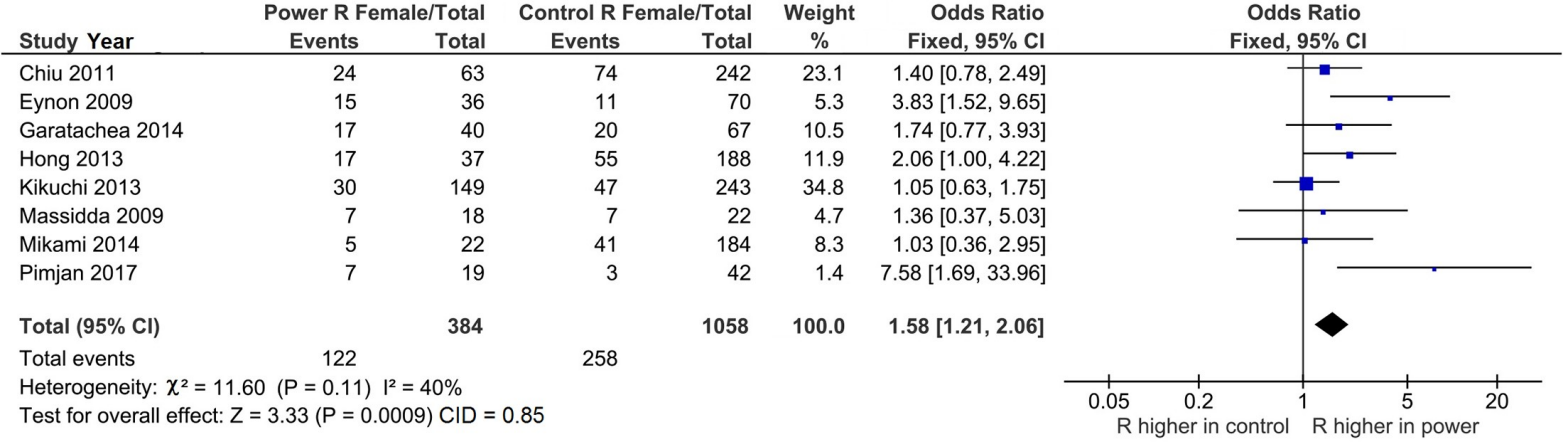
Forest plot of the R allele female effects in the post-outlier analysis. R: wild-type; CI: confidence interval; P: P-value; χ^2^: chi-square; CID: confidence interval difference.

Operation of outlier treatment is outlined in Figs 3–5 for the R allele comparison among females with ORs that favor MP over MC. In Fig 3, the pooled effect (OR 1.39, 95% CI 0.93–2.08) was not significant (P^a^ = 0.11) and heterogeneous (P^b^ = 0.003, I^2^ = 63%). The sources of this heterogeneity were identified [30,51] with the Galbraith plot (Fig 4). Fig 5 shows the PSO value of acquired significance (P^a^ = 0.0009) and reduced heterogeneity (P^b^ = 0.11, I^2^ = 40%) with pooled female effect of the R allele higher in MP compared to MC (OR 1.58, 95% CI 1.21–2.06, P^a^ = 0.0009).

### Effects of outlier treatment

Table 3 presents the trends from PRO to PSO outcomes using four parameters (high significance, heterogeneity, homogeneity and precision). In the subgroups, high significance declined (20% to 9%) as did heterogeneity (73% to 27%). In the overall analysis, however, heterogeneity disappeared (100% to 0%). Outlier treatment did not appear to impact on homogeneity in all comparisons. The precision parameter (indicated by CID) shows that the PRO and PSO values had normal distributions in the overall (KS: P = 0.09–0.16) but not the subgroups (KS: P = 0.002–0.01). For both comparisons, the PSO values were less than PRO with the overall mean ± SD (0.16 ± 0.02 < 0.24 ± 0.06) and subgroup median (IQR) of 0.27 (0.22–0.77) < 0.36 (0.28–0.91). However, the CID differences were not significant in the overall analysis (t = 2.30, P = 0.08) and the subgroups (U = 47.50, P = 0.41). Despite their non-significance, these differences suggest increase in precision.

**Table 3:** Effects of outlier treatment in power sports outcomes.

		Overall		Subgroups	
Parameter	Indicator	PRO n/Total (%)	PSO n/Total (%)	PRO n/Total (%)	PSO n/Total (%)
High significance	P < 10^−5^	0/3 (0)	0/3 (0)	3/15 (20)	1/11 (9)
Heterogeneity	Random-effects	3/3 (100)	0/3 (0)	11/15 (73)	3/11 (27)
Homogeneity	I^2^ = 0%	0/3 (0)	0/3 (0)	3/11 (20)	2/11 (18)
Precision **	CID*	0.24 ± 0.06	0.16 ± 0.02	0.36 (0.28–0.91)	0.27 (0.22–0.77)

PRO: pre-outlier; PSO: post-outlier; CID: confidence interval difference;

* mean ± standard deviation for overall; median (interquartile range) for subgroups;

** CID comparison between PRO and PSO in overall (t-test: t = 2.30, P = 0.08) and subgroups (Mann-Whitney U test: U = 47.50, P = 0.41)

### Tests of interaction

Interaction tests were performed on the significant R allele and RX genotype subgroup outcomes. Table 4 shows that of the 5 comparisons subjected to these tests, only the significant female effect (OR 1.58, P = 0.0009) compared with that of the non-significant male effect (OR 1.06, P = 0.31) resulted in significant interaction (P_interaction BC_ = 0.03) suggesting improved association. This comparative outcome strengthens the statistical evidence favoring the MP female effect.

**Table 4:** Tests of interaction.

Outlier status	Genetic component	a		b	OR^a^	v	OR^b^	Uncorrected P_interaction_	Corrected P_interaction BC_
PRO	R	Western	v	Asian	**1.11**	v	0.83	0.30	>1
PSO	R	Western	v	Asian	**1.12**	v	1.11	0.97	>1
PRO	R	Western	v	African	**1.11**	v	1.03	0.64	>1
PSO	RX	Asian	v	Western	**1.91**	v	1.03	**0.012**	0.06
PSO	R	Female	v	Male	**1.58**	v	1.06	**0.007**	**0.03**

PRO: pre-outlier; PSO: post-outlier; R: common allele; RX: heterozygous genotype; v: versus; OR: odds ratio; a: subgroup with significant ORs; b: subgroup with non-significant ORs; P-values were Bonferroni-corrected (_BC_). Values in bold indicate significance (P < 0.05) of associations (under OR^a^) and P-values (corrected and uncorrected)

### Sensitivity analysis

In this section, we focused only the significant MP pooled ORs. Table 5 shows robust outcomes in the following: (i) more in PSO than in PRO; (ii) in the overall analyses regardless of outlier status; (iii) in the subgroups, only race in PSO and in gender, only female and (iv) as to genetic component, in the R allele more than in the RX genotype.

**Table 5:** Sensitivity analysis.

Comparison	Genetic component	Outlier status	Sensitivity outcome
Overall	R	PRO	Robust
Overall	R	PSO	Robust
Western	R	PRO	[4,52]
Western	R	PSO	Robust
Asian	RX	PSO	Robust
Male	RX	PSO	[30,53–57]
Female	R	PSO	Robust

R: common allele; RX: heterozygous genotype; PRO: pre-outlier; PSO: post-outlier; presence of reference number indicates intervening studies

### Publication bias

Study-specific ORs under each of R and RX genetic components showed the non-normal distribution of the operating data (KS test: P = 0.002–0.02). Given this data distribution, we used the Begg-Mazumdar correlation [36] to assess publication bias. We applied this test on comparisons with > 10 studies [58] and those with significant outcomes only. Table 6 shows no evidence of publication bias in all comparisons (P > 0.05).

**Table 6:** Test for publication bias.

				Begg Mazumdar correlation test	
Group/subgroup	Genetic component	Outlier status	n	Kendall τ	P-value
Overall	R	PRO	44	-0.08	0.45
Overall	R	PSO	38	-0.10	0.39
Western	R	PRO	31	-0.03	0.84
Western	R	PSO	28	-0.09	0.52
Male	RX	PSO	20	-0.05	0.75

R: common allele; RX: heterozygous genotype; PRO: pre-outlier; PSO: post-outlier; n: number of studies

## Discussion

This meta-analysis has two principal findings: (i) overall associations of the R allele and RX genotype with PS performance were validated with outlier and subgroup treatments and (ii) the R allele was associated with Westerns and females whereas the RX genotype was associated with Asians and males. Gender-wise, the R allele effects differed between males and females. The significant Asian and male/female outcomes were the results of outlier treatment. This treatment impacted on heterogeneity (reduced or eliminated) of the gender outcomes, but not the Asian outcomes. Thus, retention of heterogeneity in the PSO Asian pooled effects warrants caution in its interpretation.

A total sample size of 20,753 individuals for this meta-analysis seems large until this number is contextualized in terms of the controls (n = 15,903) which is > 3X those of the athletes (n = 4,850). In the Western subgroup of Wang et al [22], there were 13 controls for every 1 athlete participant (n = 1,694/130). The gap between large numbers of controls and few elite athlete participants reduces statistical power. This then may be one reason why genetic association studies of elite sports performance have low statistical power [8]. In view of this limitation, the meta-analysis approach, with its aggregate sample sizes may be suitable for examining association of *ACTN3* R577X with PS performance.

### Significance of the findings

The *ACTN3* R577X polymorphism has a repeated influence on elite athletic performance [4, 57]. This is why improved levels of evidence need to be presented from several front-line studies. The association between the *ACTN3* R577X polymorphism on elite PS performance has been established across studies that have employed differing methodologies. Our findings of RR and RX associations with PS appear to agree with both human [59] and animal studies [60]. Compared with X allele carriers, studies have shown that RR genotype and R allele carriers may have more muscle size and strength [7,61,62], faster sprint times [63] and a higher proportion of fast-twitch muscle fibers [59,64]. Likewise, PS athletes have been reported to have a higher frequency of the RR + RX genotype (presence of α-actinin-3 protein) in their fast-twitch skeletal muscle compared to controls [4]. However, not all studies agree with these findings [25,65–68]. Nevertheless, the importance of *ACTN3* in muscle structure and phenotype [63,69] directly affects elite PS performance. In our study, marginalizing the role of the X allele in the PS phenotype was based on their control-leaning (MC) and/or null effects as well as non-significance which warranted a neutral position as to its role in PS. We maintain this neutrality, despite the suggestion that the XX genotype is detrimental for elite sprint and power performance [70].

### Subgroup significance

Differential findings delineated the associations between the races. Outcomes in Africans were marginalized in view of their non-associative (null/MC) effects across comparisons. Conversely, there were significant and associative effects in Westerners (R allele in PRO and PSO) and Asians (RX genotype in PSO) and these formed the core of our subgroup findings. The RR and RX associations found in our study seem to agree with studies of Japanese and Caucasian (Western) PS athletes with higher frequencies of RR and RX than controls [4,23,53]. On the protein level, presence of *α-actinin-3* in fast-twitch skeletal muscle fibers (RR and RX genotypes at the gene level), was associated with PS performance in elite Japanese athletes [27]. Not only do the RR and RX genotypes have associations with the PS phenotype across race lines, they also cross athletic status as well, with incremental frequencies from regional to national to international levels for PS athletes [4,55,71].

Gender analysis of the R allele shows significance in females but not in males indicating the lack of support for PS in men, but with validated outcome in women. This R allele gender difference seems at odds with the men’s outcome that did not show enrichment for the RR genotype but did for the RX. Our significant male RX finding appears to contrast with the primary study of Ruiz et al. [56] who reported absence of heterozygous advantage for Spanish elite male athletes. Reasons for this disparity may be methodological which include limited statistical power, hidden confounding factors, and/or misdefined phenotypes [32]. The R allele/RR genotype appears to influence relative peak power in Japanese male athletes [27] and impact upon elite status among Taiwanese female sprint swimmers [70]. Regarding response to exercise training, women with the RR genotype (compared to XX genotype carriers) had lower muscle leg power initially but had greater increases after strength training [12].

### *ACTN3* and previous meta-analyses

A recent 2018 publication (Garton et al) on the effect of the *ACTN3* gene on skeletal muscle performance included a meta-analysis that evaluated gene dosage effects using the Bayesian random-effects model [50]. Because this statistical approach diverges from ours, we compare from the viewpoints of study number differences and genetic outcomes. From a total of 12 studies, they found a homozygote effect (R allele) of 1.4-fold, while our overall R allele outcome was 1.2-fold from 44 studies (PRO). They also reported substantial heterogeneity in the heterozygote RX effect (OR 0.98). Our overall PRO RX effect (OR = 0.90) was also heterogeneous (I^2^ = 62%). However, our outlier results yielded fixed-effects outcomes (PSO) for the R allele and RX genotype which addressed and resolved the heterogeneity issue. Garton et al [50] found that race and gender were unlikely variables that explain homozygote (R) and dominant (RX) effects. In contrast, we delineated race and gender effects with subgroup analysis. They concluded that no single genetic model explains the association between *ACTN3* and PS [50]. In contrast, our use of allele-genotype modeling yielded clear *ACTN3* R allele and RX genotype associations with PS.

Because similar methodologies were used in the 3 previous meta-analyses [10,13,49], we are able to systematically compare each of their findings with ours (S6 Table). Weyerstraß et al [49] and Alfred et al [13] presented results exclusively from examining PS performance, while Ma et al [10] pooled data from power, endurance and mixed sports. S6 Table compares the overall outcomes based on the R allele and shows where meta-analysis treatments were applied and where they were not. Our use of outlier treatment facilitated a shift from PRO to PSO rendering changes in significance (elevated or gained) and heterogeneity (reduced or eliminated). Despite these dynamic shifts being unaddressed in the previous meta-analyses, our overall pooled OR is similar to those of Ma et al [10] and Alfred et al [13] but not Weyerstraß et al [49]. In the race subgroups, the *ACTN3* R577X genotype was associated with PS performance among Europeans (Westerns) in Alfred et al [13] but not Ma et al [10] and Weyerstraß et al [49]. All three meta-analyses showed no such associations in Asians and/or African athletes.

### Comparisons with other studies

The hypothesis that the *ACTN3* R allele may confer some advantage in PS is supported by cross-sectional studies in elite athletes and non-athletes as well as mouse models of *ACTN3* deficiency [2,60,72,73]. In *ACTN3* knockout mice, the loss-of-function variant for the XX genotype resulted in eliminated expression of the *α-actinin-3* protein [60,69,74]. Such deficiency has been shown to reduce fast-twitch muscle fiber diameter, muscle mass and strength. Moreover, studies of athletes with the RR or RX genotype are associated with high muscle strength and power compared to those with the XX genotype [7]. This appears concordant with our R allele and RX genotype findings. Several case-control association studies have reported that the RR genotype is over-represented or the XX genotype is under-represented in strength and power athletes in comparison with controls [4,72].

### *ACTN3* and GWAS

The polygenic nature of gene involvement in sports performance generated studies to investigate this aspect of sports genetics. Other than haplotype and interaction studies, examining several genes simultaneously using GWAS appears to be the current fashion in genomics. In 2019, Jacques et al [75] summarized current advances in the genetics of sports performance and covered GWAS and *ACTN3* in separate sections exclusive of each other. Five years earlier (2014), a GWAS and meta-analysis study was performed on African-American and Asian (Japanese) sprint (power) athletes and identified two loci of putative interest [48] but did not include the *ACTN3* R577X polymorphism. The two PS GWAS in the literature examined grip strength and sprint but they did not include *ACTN3* which suggests the nascent status of the GWAS approach. A literature search yielded no other GWAS undertakings related to *ACTN3*, attesting to the paucity of studies in this area. Nevertheless, whole genome analysis of elite athletes has been predicted to reshape the current understanding of the genetic basis of athletic performance [76,77].

### *ACTN3* R577X effects on longevity

Beyond the prestige of elite sports performance in power rests the long term issue of skeletal muscle integrity, which is an overriding concern in a human’s lifetime, more so in the elderly. Knowledge of the genetic influence of *ACTN3* R577X on elderly populations could potentially affect health care and treatment for this segment of society. The *ACTN3* R577X genotype exhibits a potential modifying effect on muscle deterioration (sarcopenia), which is associated with aging [78]. Given the importance of resistance training in preventing and treating sarcopenia [79], the *ACTN3* R577X genotype could modify resistance training adaptations [7]. In particular, the R allele is associated with greater adaptive response to training [11] and protects against the development of sarcopenia [80]. The consensus is that the RR genotype is associated with enhanced strength and power improvements. The protective action of the RR genotype against sarcopenia is then expected among the elderly because of its potential association with better health. However, genetic data on centenarians (≥ 100 years of age) show otherwise. In two studies of Japanese and Spanish centenarians, the XX genotype frequency was reported at 23.7% and 24%, respectively, significant in the latter (P = 0.01) but not the former (P = 0.75) when compared with controls [81,82]. Moreover, frequency of the XX genotype in supercentenarians (> 110 years old) was even higher at 33% [81]. In addition, North et al [83] found that the XX genotype was associated with enhanced cardiovascular fitness. These data suggest a potential survival advantage of the XX genotype impacting old people [81]. The R allele, on the other hand, was shown to be associated with reduced frailty risk [80]. Unlike the X allele, however, the R allele is uncommon in the very old population. Thus, the inverse relationship of the R allele with longevity renders RX heterozygotes the most benefit given the advantages associated with each allele [80]. The study of Garatachea et al (2011) further complicates the *ACTN3*-longevity issue, where they found no associations of the *ACTN3* genotypes with muscle phenotypes in octogenarians [84]. In view of all these findings, associations of the two alleles (R or X) with human longevity remain complicated and their results controversial [82]. Future studies may require greater sample sizes to resolve this impasse.

### Strengths and limitations

The interpretation of our findings is best contextualized in view of its limitations and strengths. The limitations include: (i) statistical heterogeneity; (ii) possible admixtures of the Western subgroup; (iii) dominating presence of Western studies under-represented the Asian and African populations; (iv) only 8 included articles (21.1%) matched their controls with athlete participants; (v) that some included studies contain elements of endurance and mixed sports in the performance (e.g. team sports) might dilute the impact of our PS findings. On the other hand, the strengths comprise of the following: (i) confining our analysis to HWE-compliant studies effectively screened for genotyping errors, thus minimizing methodological weaknesses to the summary outputs [85]; (ii) overall methodological quality (determined by CB) of the included studies was high; (iii) 87% (33/38 articles) of the controls were defined as healthy indicating lack of heterogeneity; (iv) 71% (5/7 comparisons) of the sensitivity treatment outcomes were robust and (v) a 43% increase (from 19 studies in Weyerstraß et al [49] to 44 in this meta-analysis) in the number of included studies raised the total sample size. This enhanced the statistical power to assess associations in this study and may have minimized false-positive outcomes [42]. Our use of outlier treatment in this study also has strengths and limitations. We have shown that this treatment is an effective meta-analysis tool in addressing heterogeneity. However, the caveat of outlier treatment is that it reduces statistical power but is more impactful when the number of studies is few (e.g. < 10). We recognize that elite athlete status is a complex condition. Whilst few articles (6 and 4, respectively) mentioned gene-gene interaction and haplotypes, considerably more (20/38 articles) addressed the importance of gene-environment interplay (S2 Table).

### Practical applications

Strengthened evidence from this meta-analysis could establish the *ACTN3* gene to form part of the genetic repertoire for identifying elite athletes with PS potential. The future of genetic testing for talent identification of elite sprint and power athletes has been posited to include the *ACTN3* R577X genotype [70]. In fact, a genetic-based algorithm (which includes *ACTN3*) has been proposed to determine training response by predicting potential for athletic performance [80]. However, reaction to this proposal has been viewed as premature, warranting caution in terms of its interpretation and implementation [84]. At present, the nascent status of the influence of genetic polymorphisms in sports precludes the recommendation of genetic testing to optimize athletic performance [48].

### Conclusions

Focusing on a single but notable polymorphism (*ACTN3* R577X) affecting PS performance [1] has allowed application of a number of meta-analytical procedures which unraveled detailed associations. These procedures managed to uncover findings that were not obvious in the component studies which we hope to have augmented existing evidence for *ACTN3* R577X associations with PS status. Varying depths of supporting evidence are important in replicating significant results [8]. The MP overall effect of the R allele in *ACTN3* R577X may be modest, but its replicative behavior observed in the subgroups likely establishes its role in enhancing PS performance.

## Supporting information

S1 List Excluded studies. (DOCX)

S1 Table Excluded studies based on HWE. (DOCX)

S2 Table Included studies. (DOCX)

S3 Table Quantitative data of *ACTN3* R577X polymorphism studies. (DOCX)

S4 Table PRISMA checklist. (DOCX)

S5 Table Meta-analysis of genetic association checklist. (DOCX)

S6 Table Comparisons with other meta-analyses. (DOCX)

## Abbreviations

[R]: Reference number
A: Alfred et al.
a: Subgroup with significant ORs
*ACTN3*: *alpha-actinin-3*
AfAm: African American
AM: Analysis model
b: Subgroup with non-significant ORs
CB: Clark-Baudouin
CI: Confidence interval
CID: Confidence interval difference
EH: Eliminated heterogeneity
ES: Elevated significance
F: Fixed-effects
GE: Gene-environment interaction
GG: Gene-gene interaction
GS: Gain in significance
GWAS: Genome-Wide Association Studies
HC: Healthy control
HP: Haplotype
HWE: Hardy-Weinberg Equilibrium
I^2^: Measure of inconsistency expressed in %
IQR: Interquartile range
K: Number designation of the study
KS: Kolmogorov-Smirnov test
Log OR: Logarithm of OR
M: Ma et al
maf: Minor allele frequency
MC: More in control
MP: More in power
n: Number of studies
NC: No change in heterogeneity
NM: No mention
OR: Odds ratio
P: P-value
P^a^: P-value for association
P^b^: P-value for heterogeneity
P_BC_: Bonferroni-corrected P-value
PRISMA: Preferred Reporting Items for Systematic Reviews and Meta-Analyses
PRO: Pre-outlier
PS: Power sports
PSO: Post-outlier
R: Wild-type *ACTN3* R577X polymorphism
RE: Random-effects
RH: Reduced heterogeneity
RNS: Retained non-significance
RS: Retained significance
RX: Heterozygous genotype of *ACTN3* R577X polymorphism
SigMP: Significantly more in power
USA: United States of America
v: versus
W: Western
WY: Weyerstraß et al
X: Variant type *ACTN3* R577X polymorphism
τ^2^: Tau-square
χ^2^: Chi-square
