# Supplementary material for: Association of the ACTN3 R577X (rs1815739) polymorphism with elite power sports: A meta-analysis
Source: PLoS One. 2019 May 30;14(5):e0217390. doi: 10.1371/journal.pone.0217390 (PMC6542526; doi:10.1371/journal.pone.0217390)
Supplement: S1 List — (DOCX) [file pone.0217390.s001.docx]

**S1 List Excluded studies**

**No control /No genotype/unsuitable data (n = 24)**

1. Bosnyak E, Trajer E, Udvardy A, Komka Z, Protzner A, Kovats T, et al. ACE and ACTN3 genes polymorphisms among female Hungarian athletes in the aspect of sport disciplines. Acta physiologica Hungarica. 2015;102(4):451-8. doi: 10.1556/036.102.2015.4.12. PubMed PMID: 26690037.

2. Coelho D PE, Rosse I, Veneroso C, Becker L, Carvalho MR, Pussieldi G, Silami-Garcia E. The alpha-actinin-3 R577X polymorphism and physical performance in soccer players. The Journal of sports medicine and physical fitness. 2015;56(3):1-24.

3. Contro V, Schiera G, Abbruzzo A, Bianco A, Amato A, Sacco A, et al. An innovative way to highlight the power of each polymorphism on elite athletes phenotype expression. European journal of translational myology. 2018;28(1):7186. doi: 10.4081/ejtm.2018.7186. PubMed PMID: 29686814; PubMed Central PMCID: PMC5895983.

4. Dionisio TJ, Thiengo CR, Brozoski DT, Dionisio EJ, Talamoni GA, Silva RB, et al. The influence of genetic polymorphisms on performance and cardiac and hemodynamic parameters among Brazilian soccer players. Applied physiology, nutrition, and metabolism = Physiologie appliquee, nutrition et metabolisme. 2017;42(6):596-604. doi: 10.1139/apnm-2016-0608. PubMed PMID: 28177711.

5. Durmic TS, Zdravkovic MD, Djelic MN, Gavrilovic TD, Djordjevic Saranovic SA, Plavsic JN, et al. Polymorphisms in ACE and ACTN3 Genes and Blood Pressure Response to Acute Exercise in Elite Male Athletes from Serbia. The Tohoku journal of experimental medicine. 2017;243(4):311-20. doi: 10.1620/tjem.243.311. PubMed PMID: 29269700.

6. Eynon N, Alves AJ, Yamin C, Sagiv M, Duarte JA, Oliveira J, et al. Is there an ACE ID - ACTN3 R577X polymorphisms interaction that influences sprint performance? International journal of sports medicine. 2009;30(12):888-91. doi: 10.1055/s-0029-1238291. PubMed PMID: 20013558.

7. Eynon N, Alves AJ, Meckel Y, Yamin C, Ayalon M, Sagiv M, et al. Is the interaction between HIF1A P582S and ACTN3 R577X determinant for power/sprint performance? Metabolism: clinical and experimental. 2010;59(6):861-5. doi: 10.1016/j.metabol.2009.10.003. PubMed PMID: 20005538.

8. Galeandro V, Notarnicola A, Bianco A, Tafuri S, Russo L, Pesce V, et al. ACTN3/ACE genotypes and mitochondrial genome in professional soccer players performance. Journal of biological regulators and homeostatic agents. 2017;31(1):207-13. PubMed PMID: 28337894.

9. Gentil P, Pereira RW, Leite TK, Bottaro M. ACTN3 R577X Polymorphism and Neuromuscular Response to Resistance Training. Journal of sports science & medicine. 2011;10(2):393-9. PubMed PMID: 24149888; PubMed Central PMCID: PMC3761844.

10. Gomez-Gallego F, Santiago C, Gonzalez-Freire M, Muniesa CA, Fernandez Del Valle M, Perez M, et al. Endurance performance: genes or gene combinations? International journal of sports medicine. 2009;30(1):66-72. doi: 10.1055/s-2008-1038677. PubMed PMID: 18651373.

11. Hanson ED, Ludlow AT, Sheaff AK, Park J, Roth SM. ACTN3 genotype does not influence muscle power. International journal of sports medicine. 2010;31(11):834-8. doi: 10.1055/s-0030-1263116. PubMed PMID: 20830656.

12. Itaka T TY, Inoue K, Agemizu K, Aruga S, Machida S. ACTN3 R577X gene polymorphism may play a role to determine the duration of judo matches. TRENDS in Sport Sciences. 2017;2(24):67-71.

13. Kikuchi N, Nakazato K, Min SK, Ueda D, Igawa S. The ACTN3 R577X polymorphism is associated with muscle power in male Japanese athletes. Journal of strength and conditioning research. 2015;28(7):1783-9. doi: 10.1519/JSC.0000000000000338. PubMed PMID: 24343324.

14. Kikuchi N, Zempo H, Fuku N, Murakami H, Sakamaki-Sunaga M, Okamoto T, et al. Association between ACTN3 R577X Polymorphism and Trunk Flexibility in 2 Different Cohorts. International journal of sports medicine. 2017;38(5):402-6. doi: 10.1055/s-0042-118649. PubMed PMID: 28303562.

15. Miarka B BC, Fukuda DH , Barros CC, Goulart C, Bello FD, Del Vecchio FB. Influence of ACTN3 R/X gene polymorphisms on racing strategy in rowing athletes. International Journal of Performance Analysis in Sport. 2017:1-9. doi: 10.1080/24748668.2017.1416527

16. Oliveira EC RP, Salgueirosa FM, Seniski GG, Wharton L, Osiecki R. Effect of ACTN3 R577X Genotypes on Muscle Strength and Power in Brazilian Mixed Martial Arts Athletes. Journal of the American Society of Exercise Physiologists. 2018;21(2):202-13.

17. Orysiak J, Busko K, Mazur-RoZycka J, Michalski R, Gajewski J, Malczewska-Lenczowska J, et al. Relationship Between ACTN3 R577X Polymorphism and Physical Abilities in Polish Athletes. Journal of strength and conditioning research. 2015;29(8):2333-9. doi: 10.1519/JSC.0000000000000880. PubMed PMID: 25734782.

18. Papadimitriou ID, Lucia A, Pitsiladis YP, Pushkarev VP, Dyatlov DA, Orekhov EF, et al. ACTN3 R577X and ACE I/D gene variants influence performance in elite sprinters: a multi-cohort study. BMC genomics. 2016;17:285. doi: 10.1186/s12864-016-2462-3. PubMed PMID: 27075997; PubMed Central PMCID: PMC4831144.

19. Pimenta EM, Coelho DB, Veneroso CE, Barros Coelho EJ, Cruz IR, Morandi RF, et al. Effect of ACTN3 gene on strength and endurance in soccer players. Journal of strength and conditioning research. 2013;27(12):3286-92. doi: 10.1519/JSC.0b013e3182915e66. PubMed PMID: 23539075.

20. Rodríguez-Romo G RJ, Santiago C, Fiuza-Luces C, González-Freire M, Gómez-Gallego F, Morán M, Lucia A Does the ACE I/D polymorphism, alone or in combination with the ACTN3 R577X polymorphism, influence muscle power phenotypes in young, non-athletic adults? European journal of applied physiology. 2010;110(6):1099–106 doi: 10.1007/s00421-010-1608-2.

21. Ulucan K, Gole S, Altindas N, Guney A. Preliminary Findings of alpha -Actinin-3 Gene Distribution in Elite Turkish Wind Surfers. Balkan journal of medical genetics : BJMG. 2013;16(1):69-72. doi: 10.2478/bjmg-2013-0020. PubMed PMID: 24265587; PubMed Central PMCID: PMC3835299.

22. Ulucan K, Sercan C, Biyikli T. Distribution of Angiotensin-1 Converting Enzyme Insertion/Deletion and alpha-Actinin-3 Codon 577 Polymorphisms in Turkish Male Soccer Players. Genetics & epigenetics. 2015;7:1-4. doi: 10.4137/GEG.S31479. PubMed PMID: 26448692; PubMed Central PMCID: PMC4578552.

23. Vancini RL, Pesquero JB, Fachina RJ, Andrade Mdos S, Borin JP, Montagner PC, et al. Genetic aspects of athletic performance: the African runners phenomenon. Open access journal of sports medicine. 2014;5:123-7. doi: 10.2147/OAJSM.S61361. PubMed PMID: 24891818; PubMed Central PMCID: PMC4037248.

24. Wang G, Mikami E, Chiu LL, A DEP, Deason M, Fuku N, et al. Association analysis of ACE and ACTN3 in elite Caucasian and East Asian swimmers. Medicine and science in sports and exercise. 2013;45(5):892-900. doi: 10.1249/MSS.0b013e31827c501f. PubMed PMID: 23190598.

**No power sports data (endurance and mixed) (n = 22)**

25. Ahmetov, II, Druzhevskaya AM, Astratenkova IV, Popov DV, Vinogradova OL, Rogozkin VA. The ACTN3 R577X polymorphism in Russian endurance athletes. British journal of sports medicine. 2008;44(9):649-52. doi: 10.1136/bjsm.2008.051540. PubMed PMID: 18718976.

26. Ahmetov, II, Druzhevskaya AM, Lyubaeva EV, Popov DV, Vinogradova OL, Williams AG. The dependence of preferred competitive racing distance on muscle fibre type composition and ACTN3 genotype in speed skaters. Experimental physiology. 2011;96(12):1302-10. doi: 10.1113/expphysiol.2011.060293. PubMed PMID: 21930675.

27. Doring FE, Onur S, Geisen U, Boulay MR, Perusse L, Rankinen T, et al. ACTN3 R577X and other polymorphisms are not associated with elite endurance athlete status in the Genathlete study. Journal of sports sciences. 2010;28(12):1355-9. doi: 10.1080/02640414.2010.507675. PubMed PMID: 20845221.

28. Grealy R, Herruer J, Smith CL, Hiller D, Haseler LJ, Griffiths LR. Evaluation of a 7-Gene Genetic Profile for Athletic Endurance Phenotype in Ironman Championship Triathletes. PloS one. 2015;10(12):e0145171. doi: 10.1371/journal.pone.0145171. PubMed PMID: 26716680; PubMed Central PMCID: PMC4696732.

29. Jastrzebski Z L-DA, Kolbowicz M, Tomiak T. Association of the ACTN3 R577X polymorphism in Polish rowers. BALTIC JOURNAL OF HEALTH AND PHYSICAL ACTIVITY. 2014;6(3):205-10. doi: 10.2478/bjha-2014-0019.

30. Heffernan SM, Kilduff LP, Erskine RM, Day SH, McPhee JS, McMahon GE, et al. Association of ACTN3 R577X but not ACE I/D gene variants with elite rugby union player status and playing position. Physiological genomics. 2016;48(3):196-201. doi: 10.1152/physiolgenomics.00107.2015. PubMed PMID: 26757799; PubMed Central PMCID: PMC4929273.

31. Kothari S, Chheda, P, Chawla, S, Chatterjee, L, Chaudhry, SK and Das, BR. ACTN3 R577X Polymorphism in Asian Indian Athletes. Int J Hum Genet. 2011;11(3):149-53.

32. Li YC, Wang LQ, Yi LY, Liu JH, Hu Y, Lu YF, et al. ACTN3 R577X genotype and performance of elite middle-long distance swimmers in China. Biology of sport. 2017;34(1):39-43. doi: 10.5114/biolsport.2017.63731. PubMed PMID: 28416896; PubMed Central PMCID: PMC5377559.

33. Lucia A, Gomez-Gallego F, Santiago C, Bandres F, Earnest C, Rabadan M, et al. ACTN3 genotype in professional endurance cyclists. International journal of sports medicine. 2006;27(11):880-4. doi: 10.1055/s-2006-923862. PubMed PMID: 16612741.

34. Massidda M, Corrias L, Ibba G, Scorcu M, Vona G, Calo CM. Genetic markers and explosive leg-muscle strength in elite Italian soccer players. The Journal of sports medicine and physical fitness. 2012;52(3):328-34. PubMed PMID: 22648472.

35. Muniesa CA, Gonzalez-Freire M, Santiago C, Lao JI, Buxens A, Rubio JC, et al. World-class performance in lightweight rowing: is it genetically influenced? A comparison with cyclists, runners and non-athletes. British journal of sports medicine. 2008;44(12):898-901. doi: 10.1136/bjsm.2008.051680. PubMed PMID: 18801770.

36. Orysiak J, Sitkowski D, Zmijewski P, Malczewska-Lenczowska J, Cieszczyk P, Zembron-Lacny A, et al. Overrepresentation of the ACTN3 XX genotype in elite canoe and kayak paddlers. Journal of strength and conditioning research. 2015;29(4):1107-12. doi: 10.1519/JSC.0000000000000717. PubMed PMID: 25268288.

37. Papadimitriou ID, Lockey SJ, Voisin S, Herbert AJ, Garton F, Houweling PJ, et al. No association between ACTN3 R577X and ACE I/D polymorphisms and endurance running times in 698 Caucasian athletes. BMC genomics. 2018;19(1):13. doi: 10.1186/s12864-017-4412-0. PubMed PMID: 29298672; PubMed Central PMCID: PMC5753575.

38. Paparini A, Ripani M, Giordano GD, Santoni D, Pigozzi F, Romano-Spica V. ACTN3 genotyping by real-time PCR in the Italian population and athletes. Medicine and science in sports and exercise. 2007;39(5):810-5. doi: 10.1097/mss.0b013e3180317491. PubMed PMID: 17468578.

39. Rodriguez-Romo G, Yvert T, de Diego A, Santiago C, Diaz de Durana AL, Carratala V, et al. No association between ACTN3 R577X polymorphism and elite judo athletic status. International journal of sports physiology and performance. 2013;8(5):579-81. PubMed PMID: 23348074.

40. Salgueirosa FM RP, Seniski GG, Wharton L, Osiecki R. ACTN3 R577X and ACE I/D Genotype Frequencies of Professional Soccer Players in Brazil. Journal of the American Society of Exercise Physiologists. 2017;20(6):129-38.

41. Saunders CJ, September AV, Xenophontos SL, Cariolou MA, Anastassiades LC, Noakes TD, et al. No association of the ACTN3 gene R577X polymorphism with endurance performance in Ironman Triathlons. Annals of human genetics. 2007;71(Pt 6):777-81. doi: 10.1111/j.1469-1809.2006.00385.x. PubMed PMID: 17627799.

42. Shang X, Huang C, Chang Q, Zhang L, Huang T. Association between the ACTN3 R577X polymorphism and female endurance athletes in China. International journal of sports medicine. 2010;31(12):913-6. doi: 10.1055/s-0030-1265176. PubMed PMID: 20936592.

43. Silva MS, Bolani W, Alves CR, Biagi DG, Lemos JR, Jr., da Silva JL, et al. Elimination of influences of the ACTN3 R577X variant on oxygen uptake by endurance training in healthy individuals. International journal of sports physiology and performance. 2015;10(5):636-41. doi: 10.1123/ijspp.2014-0205. PubMed PMID: 25569611.

44. Tsianos GI, Evangelou E, Boot A, Zillikens MC, van Meurs JB, Uitterlinden AG, et al. Associations of polymorphisms of eight muscle- or metabolism-related genes with performance in Mount Olympus marathon runners. Journal of applied physiology. 2010;108(3):567-74. doi: 10.1152/japplphysiol.00780.2009. PubMed PMID: 20044476.

45. Yamak B YM, Bagci H, Imamoglu O. Association between Sport Performance and Alpha-Actinin-3 Gene R577X Polymorphism. Int J Hum Genet. 2015;15(1):13-9.

46. Yvert T, Miyamoto-Mikami E, Murakami H, Miyachi M, Kawahara T, Fuku N. Lack of replication of associations between multiple genetic polymorphisms and endurance athlete status in Japanese population. Physiological reports. 2016;4(20). doi: 10.14814/phy2.13003. PubMed PMID: 27798356; PubMed Central PMCID: PMC5099965.

**Not elite athletes (n = 9)**

47. Ahmetov I GD, Astratenkova IV, Druzhevskaya AM, Malinin AV, Romanova EE, Rogozkin VA. The association of ACE, ACTN3 and PPARA gene variants with strength phenotypes in middle school-age children. The Journal of Physiological Sciences. 2013;63(1):79-85.

48. Bhagi S, Srivastava S, Sarkar S, Singh SB. Distribution of performance-related gene polymorphisms (ACTN3 R577X and ACE ID) in different ethnic groups of the Indian Army. Journal of basic and clinical physiology and pharmacology. 2013;24(4):225-34. doi: 10.1515/jbcpp-2013-0068. PubMed PMID: 24114905.

49. Chiu LL, Chen TW, Hsieh SS, Hsieh LL. ACE I/D, ACTN3 R577X, PPARD T294C and PPARGC1A Gly482Ser polymorphisms and physical fitness in Taiwanese late adolescent girls. The journal of physiological sciences : JPS. 2012;62(2):115-21. doi: 10.1007/s12576-011-0189-0. PubMed PMID: 22247001.

50. Clarkson PM DJ, Gordish-Dressman H, Thompson PD, Hubal MJ, Urso M, Price TB, Angelopoulos TJ, Gordon PM, Moyna NM, Pescatello LS, Visich PS,, Zoeller RF SR, Hoffman EP. ACTN3 genotype is associated with increases in muscle strength in response to resistance training in women. J Appl Physiol. 2005;99:154-63. doi: 10.1152/japplphysiol.01139.2004.

51. Delmonico MJ, Kostek MC, Doldo NA, Hand BD, Walsh S, Conway JM, et al. Alpha-actinin-3 (ACTN3) R577X polymorphism influences knee extensor peak power response to strength training in older men and women. The journals of gerontology Series A, Biological sciences and medical sciences. 2007;62(2):206-12. PubMed PMID: 17339648.

52. Erskine RM, Williams AG, Jones DA, Stewart CE, Degens H. The individual and combined influence of ACE and ACTN3 genotypes on muscle phenotypes before and after strength training. Scandinavian journal of medicine & science in sports. 2014;24(4):642-8. doi: 10.1111/sms.12055. PubMed PMID: 23384112.

53. Kikuchi N, Yoshida S, Min SK, Lee K, Sakamaki-Sunaga M, Okamoto T, et al. The ACTN3 R577X genotype is associated with muscle function in a Japanese population. Applied physiology, nutrition, and metabolism = Physiologie appliquee, nutrition et metabolisme. 2015;40(4):316-22. doi: 10.1139/apnm-2014-0346. PubMed PMID: 25761735.

54. Santiago C, Rodriguez-Romo G, Gomez-Gallego F, Gonzalez-Freire M, Yvert T, Verde Z, et al. Is there an association between ACTN3 R577X polymorphism and muscle power phenotypes in young, non-athletic adults? Scandinavian journal of medicine & science in sports. 2010;20(5):771-8. doi: 10.1111/j.1600-0838.2009.01017.x. PubMed PMID: 19807896.

55. Shang X, Zhang F, Zhang L, Huang C. ACTN3 R577X polymorphism and performance phenotypes in young Chinese male soldiers. Journal of sports sciences. 2012;30(3):255-60. doi: 10.1080/02640414.2011.619203. PubMed PMID: 22224919.

**Duplicate data (n = 6)**

56. Eynon N, Ruiz JR, Femia P, Pushkarev VP, Cieszczyk P, Maciejewska-Karlowska A, et al. The ACTN3 R577X polymorphism across three groups of elite male European athletes. PloS one. 2012;7(8):e43132. doi: 10.1371/journal.pone.0043132. PubMed PMID: 22916217; PubMed Central PMCID: PMC3420864.

57. Ginevičienė V PE, Milašius K, Kučinskas V. Gene variants related to the power performance of the Lithuanian athletes. Central European Journal of Biology. 2011;6(1):48-57. doi: 10.2478/s11535-010-0102-5.

58. Grealy R, Smith CL, Chen T, Hiller D, Haseler LJ, Griffiths LR. The genetics of endurance: frequency of the ACTN3 R577X variant in Ironman World Championship athletes. Journal of science and medicine in sport. 2013;16(4):365-71. doi: 10.1016/j.jsams.2012.08.013. PubMed PMID: 23092649.

59. Kikuchi N, Min SK, Ueda D, Igawa S, Nakazato K. Higher frequency of the ACTN3 R allele + ACE DD genotype in Japanese elite wrestlers. Journal of strength and conditioning research. 2012;26(12):3275-80. doi: 10.1519/JSC.0b013e318273679d. PubMed PMID: 22996021.

60. Ribas MR ONZ, Salgueirosa F, Fernandes P, Matos O, Bassan JC. Association of actn3 R577X and ACE I/D polymorphisms in Brazilians wrestlers. Revista Brasileira de Medicina do Esporte. 2017;23(6):469-72. doi: 10.1590/1517-869220172306171864

61. Yang R, Shen X, Wang Y, Voisin S, Cai G, Fu Y, et al. ACTN3 R577X Gene Variant Is Associated With Muscle-Related Phenotypes in Elite Chinese Sprint/Power Athletes. Journal of strength and conditioning research. 2017;31(4):1107-15. doi: 10.1519/JSC.0000000000001558. PubMed PMID: 27442335.
