# Supplementary material for: Association of the ACTN3 R577X (rs1815739) polymorphism with elite power sports: A meta-analysis
Source: PLoS One. 2019 May 30;14(5):e0217390. doi: 10.1371/journal.pone.0217390 (PMC6542526; doi:10.1371/journal.pone.0217390)
Supplement: S1 Table — (DOCX) [file pone.0217390.s002.docx]

**S1 Table Articles whose controls deviated from the HWE**

| K | First author | Country | Year | maf | HWE P-value | [R] |
| --- | --- | --- | --- | --- | --- | --- |
| 1 | Ben-Zaken | Israel | 2015 | 0.482 | 0.005 | [[1](#_ENREF_1)] |
| 2 | Djarova | Zulu | 2011 | 0.100 | 0.047 | [[2](#_ENREF_2)] |
| 3 | Egorova | Russia | 2014 | 0.396 | 0.029 | [[3](#_ENREF_3)] |
| 4 | Grenda | Poland | 2014 | 0.367 | 0.046 | [[4](#_ENREF_4)] |
| 5 | Guilherme | Brazil | 2018 | 0.440 | 0.031 | [[5](#_ENREF_5)] |
| 6 | Gunel | Turkey | 2014 | 0.540 | 0.000006 | [[6](#_ENREF_6)] |
| 7 | Honarpour | Iran | 2017 | 0.421 | 0.00001 | [[7](#_ENREF_7)] |
| 8 | Massidda | Italy | 2015 | 0.410 | 0.008 | [[8](#_ENREF_8)] |
| 9 | Persi | Italy | 2013 | 0.490 | 0.00005 | [[9](#_ENREF_9)] |
|  |  |  |  |  |  |  |

K: number designation of the study; maf: minor allele frequency; HWE: Hardy-Weinberg Equilibrium; [R]: reference number

**References:**

1. Ben-Zaken S, Eliakim A, Nemet D, Rabinovich M, Kassem E, Meckel Y. ACTN3 Polymorphism: Comparison Between Elite Swimmers and Runners. Sports medicine - open. 2015;1(1):13. doi: 10.1186/s40798-015-0023-y.

2. Djarova T, Watson, G., Basson, A., Grace, J., Cloete, J. and Ramakoaba, A. ACTN3 and TNF gene polymorphism association with C-reactive protein, uric acid, lactate and physical characteristics in young African cricket players. African J Biochem Res. 2011;5(1):22-7.

3. Egorova ES, Borisova AV, Mustafina LJ, Arkhipova AA, Gabbasov RT, Druzhevskaya AM, et al. The polygenic profile of Russian football players. Journal of sports sciences. 2014;32(13):1286-93. doi: 10.1080/02640414.2014.898853.

4. Grenda A, Leonska-Duniec A, Kaczmarczyk M, Ficek K, Krol P, Cieszczyk P, et al. Interaction Between ACE I/D and ACTN3 R557X Polymorphisms in Polish Competitive Swimmers. Journal of human kinetics. 2014;42:127-36. doi: 10.2478/hukin-2014-0067.

5. Guilherme J, Bertuzzi R, Lima-Silva AE, Pereira ADC, Lancha Junior AH. Analysis of sports-relevant polymorphisms in a large Brazilian cohort of top-level athletes. Annals of human genetics. 2018. doi: 10.1111/ahg.12248.

6. Gunel T, Gumusoglu E, Hosseini MK, Yilmazyildirim E, Dolekcap I, Aydinli K. Effect of angiotensin I-converting enzyme and alpha-actinin-3 gene polymorphisms on sport performance. Molecular medicine reports. 2014;9(4):1422-6. doi: 10.3892/mmr.2014.1974.

7. Honarpour A, Mohseni, M, Hajiagha, SG, Irani, S, Najmabadi, H. Investigation of the Relationship Between a Genetic Polymorphism in ACTN3 and Elite Sport Performance Among Iranian Soccer Players. Iranian Rehabilitation Journal. 2017;15(2):149-54.

8. Massidda M, Bachis V, Corrias L, Piras F, Scorcu M, Culigioni C, et al. ACTN3 R577X polymorphism is not associated with team sport athletic status in Italians. Sports medicine - open. 2015;1(1):6. doi: 10.1186/s40798-015-0008-x.

9. Persi A, Maltese PE, Bertelli M, Cecchin S, Ciaghi M, Guarnieri MC, et al. Polymorphisms of alpha-actinin-3 and ciliary neurotrophic factor in national-level Italian athletes. Panminerva medica. 2013;55(2):217-24.
