# Supplementary material for: Association of the ACTN3 R577X (rs1815739) polymorphism with elite power sports: A meta-analysis
Source: PLoS One. 2019 May 30;14(5):e0217390. doi: 10.1371/journal.pone.0217390 (PMC6542526; doi:10.1371/journal.pone.0217390)
Supplement: S2 Table — (DOCX) [file pone.0217390.s003.docx]

**S2 Table Characteristics of the included articles**

| [R] | M | A | WY | G | First author | Year | Country | Race | GG | HP | GE | HC | Match | Sample source | CB |
| --- | --- | --- | --- | --- | --- | --- | --- | --- | --- | --- | --- | --- | --- | --- | --- |
| [[1](#_ENREF_1)] | -- | -- | -- | -- | Atanasov 🟋 | 2015 | Bulgaria | W | -- | -- | -- | ✔ | ✔ | blood | 9 |
| [[2](#_ENREF_2)] | -- | -- | ✔ | -- | Bell | 2012 | United Kingdom | W | -- | -- | ✔ | ✔ | NM | buccal saliva | 6 |
| [[3](#_ENREF_3)] | ✔ | -- | -- | -- | Chiu | 2011 | Taiwan | Asian | -- | -- | -- | ✔ | NM | blood | 7 |
| [[4](#_ENREF_4)] | -- | -- | ✔ | -- | Cięszczyk | 2011 | Poland | W | -- | -- | -- | ✔ | NM | buccal | 6 |
| [[5](#_ENREF_5)] | -- | -- | ✔ | -- | Cięszczyk | 2012 | Poland | W | -- | -- | -- | ✔ | NM | buccal | 7 |
| [[6](#_ENREF_6)] | ✔ | -- | -- | ✔ | Druzhevskaya | 2008 | Russia | W | -- | -- | -- | ✔ | NM | blood | 7 |
| [[7](#_ENREF_7)] | -- | -- | -- | -- | Eroğlu 🟋 | 2018 | Turkey | W | -- | -- | ✔ | ✔ | NM | blood | 4 |
| [[8](#_ENREF_8)] | ✔ | -- | ✔ | ✔ | Eynon | 2013 | Poland Russia Spain | W | -- | -- | ✔ | ✔ | ✔ | blood buccal | 7 |
| [[9](#_ENREF_9)] | -- | -- | -- | -- | Fiuza-Luces 🟋 | 2011 | Spain | W | ✔ | -- | ✔ | ✔ | ✔ | saliva | 8 |
| [[10](#_ENREF_10)] | -- | -- | ✔ | -- | Garatachea | 2014 | Spain | W | ✔ | -- | ✔ | ✔ | NM | saliva | 6 |
| [[11](#_ENREF_11)] | -- | -- | -- | -- | Gineviciene 🟋 | 2011 | Lithuania | W | -- | -- | ✔ | ✔ | NM | blood | 7 |
| [[12](#_ENREF_12)] | -- | -- | -- | -- | Gineviciene 🟋 | 2016 | Lithuania Russia | W | -- | -- | ✔ | ✔ | NM | blood | 8 |
| [[13](#_ENREF_13)] | -- | -- | -- | -- | Ginszt 🟋 | 2018 | Poland | W | -- | -- | -- | ✔ | NM | blood buccal | 6 |
| [[14](#_ENREF_14)] | -- | -- | ✔ | ✔ | Hong | 2013 | Korea | Asian | -- | -- | ✔ | ✔ | NM | blood buccal | 7 |
| [[15](#_ENREF_15)] | -- | -- | -- | -- | Innes 🟋 | 2016 | Lithuania | W | -- | -- | ✔ | ✔ | NM | blood | 8 |
| [[16](#_ENREF_16)] | -- | -- | -- | ✔ | Kikuchi | 2013 | Japan | Asian | -- | -- | ✔ | ✔ | NM | buccal | 6 |
| [[17](#_ENREF_17)] | -- | -- | ✔ | -- | Kikuchi | 2015 | Japan | Asian | -- | -- | ✔ | ✔ | NM | blood saliva | 7 |
| [[18](#_ENREF_18)] | -- | -- | -- | -- | Kim 🟋 | 2014 | Korea | Asian | -- | -- | ✔ | ✔ | NM | blood | 8 |
| [[19](#_ENREF_19)] | ✔ | -- | -- | -- | Massidda | 2009 | Italy | W | -- | -- | -- | ✔ | NM | buccal | 6 |
| [[20](#_ENREF_20)] | -- | -- | -- | -- | Mikami 🟋 | 2014 | Japan | Asian | -- | -- | -- | ✔ | NM | blood saliva | 5 |
| [[21](#_ENREF_21)] | ✔ | -- | -- | ✔ | Niemi | 2005 | Finland | W | -- | ✔ | -- | NM | NM | blood | 3 |
| [[22](#_ENREF_22)] | -- | -- | -- | -- | Orysiak 🟋 | 2014 | Poland | W | ✔ | -- | ✔ | ✔ | NM | blood | 7 |
| [[23](#_ENREF_23)] | ✔ | ✔ | -- | ✔ | Papadimitriou | 2008 | Greece | W | -- | -- | -- | NM | ✔ | blood | 6 |
| [[24](#_ENREF_24)] | -- | -- | -- | -- | Peplonska 🟋 | 2017 | Poland | W | -- | -- | ✔ | ✔ | NM | blood | 7 |
| [[25](#_ENREF_25)] | ✔ | ✔ | -- | -- | Roth | 2008 | USA | W/Black | -- | -- | -- | ✔ | NM | blood | 6 |
| [[26](#_ENREF_26)] | ✔ | -- | -- | -- | Ruiz | 2009 | Spain | W | ✔ | ✔ | ✔ | ✔ | ✔ | blood saliva | 6 |
| [[27](#_ENREF_27)] | ✔ | -- | -- | -- | Ruiz | 2011 | Spain | W | ✔ | -- | ✔ | ✔ | NM | saliva | 6 |
| [[28](#_ENREF_28)] | -- | -- | ✔ | -- | Ruiz | 2013 | Spain | W | -- | -- | -- | ✔ | NM | blood buccal | 7 |
| [[29](#_ENREF_29)] | -- | ✔ | -- | -- | Santiago | 2007 | Spain | W | -- | -- | -- | ✔ | NM | blood | 6 |
| [[30](#_ENREF_30)] | -- | ✔ | -- | -- | Saunders | 2007 | South Africa | W | ✔ | -- | ✔ | ✔ | ✔ | blood | 7 |
| [[31](#_ENREF_31)] | ✔ | ✔ | -- | ✔ | Scott | 2010 | Jamaica USA | AfAm | -- | -- | ✔ | NM | NM | buccal | 5 |
| [[32](#_ENREF_32)] | ✔ | -- | -- | -- | Sessa | 2011 | Italy | W | -- | -- | -- | ✔ | ✔ | blood | 7 |
| [[33](#_ENREF_33)] | -- | -- | -- | -- | Wang 🟋 | 2013 | Europe Asia | W/Asian | -- | ✔ | -- | ✔ | NM | blood buccal | 8 |
| [[34](#_ENREF_34)] | -- | -- | -- | -- | Wessner 🟋 | 2016 | Austria | W | -- | -- | -- | ✔ | NM | saliva | 8 |
| [[35](#_ENREF_35)] | ✔ | ✔ | ✔ | ✔ | Yang | 2003 | Australia | W | -- | -- | ✔ | ✔ | NM | blood | 7 |
| [[36](#_ENREF_36)] | -- | -- | -- | -- | Yang 🟋 | 2007 | Nigeria | African | -- | ✔ | ✔ | NM | NM | buccal | 8 |
| [[37](#_ENREF_37)] | -- | -- | -- | -- | Yang 🟋 | 2017 | China | Asian | -- | -- | -- | ✔ | NM | saliva | 8 |
| [[38](#_ENREF_38)] | -- | -- | -- | -- | Yusof 🟋 | 2016 | Malaysia | Asian | -- | -- | -- | NM | ✔ | saliva | 7 |

[R]: reference number; M: Ma et al; A: Alfred et al; WY: Weyerstraβ et al; G: Garton et al; 🟋: new study; USA: United States of

America ; AfAm: African-American; W: Western; GG: gene-gene interaction; HP: haplotype; GE: gene-environment interaction; HC:

healthy controls; CB: Clark-Baudouin; NM: no mention; Note: Eynon et al [[39](#_ENREF_39)] and Pimjan et al [[40](#_ENREF_40)] were used in gender subgroup

analysis only

**Reference list of included articles**

1. Atanasov P DT, Kalinski M, Petrov L, Kaneva R, Mugandani S, Watson G, Jemni M. ACTN3 and AMPD1 Polymorphism and Genotype Combinations in Bulgarian Athletes Performing Wingate Test. Journal of sports sciences. 2015;3:1-10. doi: 10.17265/2332-7839/2015.01.001.

2. Bell W, Colley JP, Evans WD, Darlington SE, Cooper SM. ACTN3 genotypes of Rugby Union players: distribution, power output and body composition. Annals of human biology. 2012;39(1):19-27. doi: 10.3109/03014460.2011.632648.

3. Chiu LL, Wu YF, Tang MT, Yu HC, Hsieh LL, Hsieh SS. ACTN3 genotype and swimming performance in Taiwan. International journal of sports medicine. 2011;32(6):476-80. doi: 10.1055/s-0030-1263115.

4. Cieszczyk P, Eider J, Ostanek M, Arczewska A, Leonska-Duniec A, Sawczyn S, et al. Association of the ACTN3 R577X Polymorphism in Polish Power-Orientated Athletes. Journal of human kinetics. 2011;28:55-61. doi: 10.2478/v10078-011-0022-0.

5. Cieszczyk P SM, Maciejewska-Karlowska A, Ficek K. ACTN3 R577X polymorphism in top-level Polish rowers. J Exercise Sci & Fitness. 2012;10:12-5. doi: <http://dx.doi.org/10.1016/j.jesf.2012.04.003>.

6. Druzhevskaya AM, Ahmetov, II, Astratenkova IV, Rogozkin VA. Association of the ACTN3 R577X polymorphism with power athlete status in Russians. European journal of applied physiology. 2008;103(6):631-4. doi: 10.1007/s00421-008-0763-1.

7. Eroglu O, Zileli R, Nalbant MA, Ulucan K. Prevalence of alpha actinin-3 gene (ACTN3) R577X and angiotensin converting enzyme (ACE) insertion / deletion gene polymorphisms in national and amateur Turkish athletes. Cell Mol Biol (Noisy-le-grand). 2018;64(5):24-8.

8. Eynon N, Banting LK, Ruiz JR, Cieszczyk P, Dyatlov DA, Maciejewska-Karlowska A, et al. ACTN3 R577X polymorphism and team-sport performance: a study involving three European cohorts. Journal of science and medicine in sport. 2013;17(1):102-6. doi: 10.1016/j.jsams.2013.02.005.

9. Fiuza-Luces C, Ruiz JR, Rodriguez-Romo G, Santiago C, Gomez-Gallego F, Yvert T, et al. Are 'endurance' alleles 'survival' alleles? Insights from the ACTN3 R577X polymorphism. PloS one. 2011;6(3):e17558. doi: 10.1371/journal.pone.0017558.

10. Garatachea N, Verde Z, Santos-Lozano A, Yvert T, Rodriguez-Romo G, Sarasa FJ, et al. ACTN3 R577X polymorphism and explosive leg-muscle power in elite basketball players. International journal of sports physiology and performance. 2014;9(2):226-32. doi: 10.1123/ijspp.2012-0331.

11. Gineviciene V, Pranculis A, Jakaitiene A, Milasius K, Kucinskas V. Genetic variation of the human ACE and ACTN3 genes and their association with functional muscle properties in Lithuanian elite athletes. Medicina. 2011;47(5):284-90.

12. Gineviciene V, Jakaitiene A, Aksenov MO, Aksenova AV, Druzhevskaya AM, Astratenkova IV, et al. Association analysis of ACE, ACTN3 and PPARGC1A gene polymorphisms in two cohorts of European strength and power athletes. Biology of sport. 2016;33(3):199-206. doi: 10.5604/20831862.1201051.

13. Ginszt M, Michalak-Wojnowska M, Gawda P, Wojcierowska-Litwin M, Korszen-Pilecka I, Kusztelak M, et al. ACTN3 Genotype in Professional Sport Climbers. Journal of strength and conditioning research. 2018;32(5):1311-5. doi: 10.1519/JSC.0000000000002457.

14. Hong SS, Jin HJ. Assessment of association of ACTN3 genetic polymorphism with Korean elite athletic performance. Genes Genom. 2013. doi: <http://dx.doi.org/10.1007/s13258-013-0111-7>.

15. Innes A. Association of Variation in ACTN3, MYOZ2 and MYOZ3 with Complex Quantitative Performance Phenotypes in Lithuanian Athletes and Controls: University of Stirling; 2016.

16. Kikuchi N, Ueda D, Min SK, Nakazato K, Igawa S. The ACTN3 XX genotype's underrepresentation in Japanese elite wrestlers. International journal of sports physiology and performance. 2013;8(1):57-61.

17. Kikuchi N, Miyamoto-Mikami E, Murakami H, Nakamura T, Min SK, Mizuno M, et al. ACTN3 R577X genotype and athletic performance in a large cohort of Japanese athletes. European journal of sport science. 2015;16(6):694-701. doi: 10.1080/17461391.2015.1071879.

18. Kim H, Song KH, Kim CH. The ACTN3 R577X variant in sprint and strength performance. Journal of exercise nutrition & biochemistry. 2014;18(4):347-53. doi: 10.5717/jenb.2014.18.4.347.

19. Massidda M, Vona G, Calo CM. Association between the ACTN3 R577X polymorphism and artistic gymnastic performance in Italy. Genetic testing and molecular biomarkers. 2009;13(3):377-80. doi: 10.1089/gtmb.2008.0157.

20. Mikami E, Fuku N, Murakami H, Tsuchie H, Takahashi H, Ohiwa N, et al. ACTN3 R577X genotype is associated with sprinting in elite Japanese athletes. International journal of sports medicine. 2014;35(2):172-7. doi: 10.1055/s-0033-1347171.

21. Niemi AK, Majamaa K. Mitochondrial DNA and ACTN3 genotypes in Finnish elite endurance and sprint athletes. European journal of human genetics : EJHG. 2005;13(8):965-9. doi: 10.1038/sj.ejhg.5201438.

22. Orysiak J, Busko K, Michalski R, Mazur-Rozycka J, Gajewski J, Malczewska-Lenczowska J, et al. Relationship between ACTN3 R577X polymorphism and maximal power output in elite Polish athletes. Medicina. 2014;50(5):303-8. doi: 10.1016/j.medici.2014.10.002.

23. Papadimitriou ID, Papadopoulos C, Kouvatsi A, Triantaphyllidis C. The ACTN3 gene in elite Greek track and field athletes. International journal of sports medicine. 2008;29(4):352-5. doi: 10.1055/s-2007-965339.

24. Peplonska B, Adamczyk JG, Siewierski M, Safranow K, Maruszak A, Sozanski H, et al. Genetic variants associated with physical and mental characteristics of the elite athletes in the Polish population. Scandinavian journal of medicine & science in sports. 2017;27(8):788-800. doi: 10.1111/sms.12687.

25. Roth SM, Walsh S, Liu D, Metter EJ, Ferrucci L, Hurley BF. The ACTN3 R577X nonsense allele is under-represented in elite-level strength athletes. European journal of human genetics : EJHG. 2008;16(3):391-4. doi: 10.1038/sj.ejhg.5201964.

26. Ruiz JR, Arteta D, Buxens A, Artieda M, Gomez-Gallego F, Santiago C, et al. Can we identify a power-oriented polygenic profile? Journal of applied physiology. 2009;108(3):561-6. doi: 10.1152/japplphysiol.01242.2009.

27. Ruiz JR, Fernandez del Valle M, Verde Z, Diez-Vega I, Santiago C, Yvert T, et al. ACTN3 R577X polymorphism does not influence explosive leg muscle power in elite volleyball players. Scandinavian journal of medicine & science in sports. 2011;21(6):e34-41. doi: 10.1111/j.1600-0838.2010.01134.x.

28. Ruiz JR, Santiago C, Yvert T, Muniesa C, Diaz-Urena G, Bekendam N, et al. ACTN3 genotype in Spanish elite swimmers: no "heterozygous advantage". Scandinavian journal of medicine & science in sports. 2013;23(3):e162-7. doi: 10.1111/sms.12045.

29. Santiago C, Gonzalez-Freire M, Serratosa L, Morate FJ, Meyer T, Gomez-Gallego F, et al. ACTN3 genotype in professional soccer players. British journal of sports medicine. 2007;42(1):71-3. doi: 10.1136/bjsm.2007.039172.

30. Saunders CJ, September AV, Xenophontos SL, Cariolou MA, Anastassiades LC, Noakes TD, et al. No association of the ACTN3 gene R577X polymorphism with endurance performance in Ironman Triathlons. Annals of human genetics. 2007;71(Pt 6):777-81. doi: 10.1111/j.1469-1809.2006.00385.x.

31. Scott RA, Irving R, Irwin L, Morrison E, Charlton V, Austin K, et al. ACTN3 and ACE genotypes in elite Jamaican and US sprinters. Medicine and science in sports and exercise. 2010;42(1):107-12. doi: 10.1249/MSS.0b013e3181ae2bc0.

32. Sessa F, Chetta M, Petito A, Franzetti M, Bafunno V, Pisanelli D, et al. Gene polymorphisms and sport attitude in Italian athletes. Genetic testing and molecular biomarkers. 2011;15(4):285-90. doi: 10.1089/gtmb.2010.0179.

33. Wang G, Mikami E, Chiu LL, A DEP, Deason M, Fuku N, et al. Association analysis of ACE and ACTN3 in elite Caucasian and East Asian swimmers. Medicine and science in sports and exercise. 2013;45(5):892-900. doi: 10.1249/MSS.0b013e31827c501f.

34. Wessner BS, P.; Fail, C.; Pavic, F.; Tschan, H.; Bachl, N. Genetic polymorphisms in alpha-actinin 3 and adrenoceptor beta genes in Austrian elite athletes and healthy controls. Swiss Sports & Exercise Med. 2016;64(4):13-9.

35. Yang N, MacArthur DG, Gulbin JP, Hahn AG, Beggs AH, Easteal S, et al. ACTN3 genotype is associated with human elite athletic performance. American journal of human genetics. 2003;73(3):627-31. doi: 10.1086/377590. PubMed

36. Yang N, MacArthur DG, Wolde B, Onywera VO, Boit MK, Lau SY, et al. The ACTN3 R577X polymorphism in East and West African athletes. Medicine and science in sports and exercise. 2007;39(11):1985-8. doi: 10.1249/mss.0b013e31814844c9.

37. Yang R, Shen X, Wang Y, Voisin S, Cai G, Fu Y, et al. ACTN3 R577X Gene Variant Is Associated With Muscle-Related Phenotypes in Elite Chinese Sprint/Power Athletes. Journal of strength and conditioning research. 2017;31(4):1107-15. doi: 10.1519/JSC.0000000000001558.

38. Yusof HS, R.; Zainuddin, Z.; Rooney, K.; Munir, A.; Muhamed, C. Alpha-Actinin-3 (ACTN3) R/X Gene Polymorphism and Physical Performance of Multi-Ethnic Malaysian Population. Int J Applied Exercise Physiol. 2016;5(3):19-30.

39. Eynon N, Duarte JA, Oliveira J, Sagiv M, Yamin C, Meckel Y, et al. ACTN3 R577X polymorphism and Israeli top-level athletes. International journal of sports medicine. 2009;30(9):695-8. doi: 10.1055/s-0029-1220731.

40. Pimjan L OC, Chantratita C, Pholpramool C, Cherdrungsi P, Bangrak P, Yimlamai T. A Study on ACE, ACTN3, and VDR Genes Polymorphism in Thai Weightlifters. Walailak J Sci & Tech. 2017;14.
