# Supplementary material for: Association of the ACTN3 R577X (rs1815739) polymorphism with elite power sports: A meta-analysis
Source: PLoS One. 2019 May 30;14(5):e0217390. doi: 10.1371/journal.pone.0217390 (PMC6542526; doi:10.1371/journal.pone.0217390)
Supplement: S3 Table — (DOCX) [file pone.0217390.s004.docx]

**S3 Table Quantitative data of *ACTN3* rs studies**

|  |  |  |  |  |  |  | Genotype frequencies | | | | | |  |  |  |
| --- | --- | --- | --- | --- | --- | --- | --- | --- | --- | --- | --- | --- | --- | --- | --- |
|  |  |  |  | Sample sizes | | | Case | | | Control | | | |  |  |
|  |  | First author | Year | Case | Control | Total | RR | RX | XX | RR | RX | XX | maf | HWE |  |
|  |  |  |  |  |  |  |  |  |  |  |  |  |  |  |  |
| 1 |  | Atanasov | 2015 | 52 | 109 | 161 | 20 | 24 | 8 | 45 | 50 | 14 | 0.358 | 0.985 |  |
| 2 |  | Bell | 2012 | 102 | 110 | 212 | 30 | 55 | 17 | 37 | 53 | 20 | 0.423 | 0.893 |  |
| 3 |  | Chiu | 2011 | 168 | 603 | 771 | 63 | 77 | 28 | 191 | 294 | 118 | 0.439 | 0.799 |  |
| 4 |  | Cieszczyk | 2011 | 158 | 254 | 412 | 71 | 77 | 10 | 89 | 125 | 40 | 0.404 | 0.722 |  |
| 5 |  | Cieszczyk | 2012 | 37 | 201 | 238 | 21 | 14 | 2 | 74 | 94 | 36 | 0.407 | 0.518 |  |
| 6 |  | Druzhevskaya | 2008 | 486 | 1,197 | 1,683 | 193 | 262 | 31 | 440 | 587 | 170 | 0.387 | 0.248 |  |
| 7 |  | Eroğlu 🟋 | 2018 | 15 | 37 | 52 | 1 | 10 | 4 | 10 | 18 | 9 | 0.486 | 0.873 |  |
| 8 |  | Eynon Poland | 2013 | 178 | 354 | 532 | 71 | 93 | 14 | 138 | 177 | 39 | 0.360 | 0.110 |  |
| 9 |  | Eynon Russia | 2013 | 82 | 111 | 193 | 33 | 39 | 10 | 39 | 46 | 26 | 0.441 | 0.093 |  |
| 10 |  | Eynon Spain | 2013 | 119 | 103 | 222 | 37 | 66 | 16 | 30 | 59 | 14 | 0.422 | 0.077 |  |
| 11 |  | Fiuza-Luces | 2011 | 63 | 283 | 346 | 30 | 23 | 10 | 90 | 142 | 51 | 0.431 | 0.699 |  |
| 12 |  | Garatachea | 2014 | 100 | 283 | 383 | 37 | 42 | 21 | 90 | 141 | 52 | 0.433 | 0.804 |  |
| 13 |  | Gineviciene | 2011 | 51 | 250 | 301 | 19 | 24 | 8 | 98 | 126 | 26 | 0.356 | 0.117 |  |
| 14 |  | Gineviciene Lithuania | 2016 | 47 | 255 | 302 | 18 | 24 | 5 | 102 | 127 | 26 | 0.351 | 0.137 |  |
| 15 |  | Gineviciene Russia | 2016 | 114 | 947 | 1,061 | 49 | 52 | 13 | 344 | 475 | 128 | 0.386 | 0.073 |  |
| 16 |  | Ginszt | 2018 | 100 | 100 | 200 | 44 | 40 | 15 | 33 | 50 | 17 | 0.420 | 0.790 |  |
| 17 |  | Hong | 2013 | 151 | 361 | 512 | 27 | 46 | 11 | 110 | 186 | 65 | 0.438 | 0.374 |  |
| 18 |  | Innes 🟋 | 2016 | 11 | 198 | 209 | 5 | 5 | 1 | 69 | 102 | 27 | 0.393 | 0.267 |  |
| -19 |  | Kikuchi | 2013 | 135 | 243 | 378 | 38 | 68 | 29 | 47 | 125 | 71 | 0.549 | 0.544 |  |
| 20 |  | Kikuchi | 2015 | 337 | 810 | 1,147 | 97 | 177 | 63 | 170 | 421 | 219 | 0.530 | 0.218 |  |
| 21 |  | Kim | 2014 | 121 | 854 | 975 | 49 | 58 | 14 | 255 | 436 | 163 | 0.446 | 0.334 |  |
| 22 |  | Massidda | 2009 | 35 | 53 | 88 | 17 | 17 | 1 | 17 | 26 | 10 | 0.434 | 0.992 |  |
| 23 |  | Mikami | 2014 | 134 | 649 | 783 | 33 | 78 | 23 | 132 | 346 | 171 | 0.530 | 0.074 |  |
| 24 |  | Niemi | 2005 | 26 | 123 | 149 | 12 | 13 | 1 | 55 | 56 | 12 | 0.325 | 0.679 |  |
| 25 |  | Orysiak | 2014 | 200 | 354 | 554 | 80 | 96 | 24 | 140 | 176 | 38 | 0.356 | 0.112 |  |
| 26 |  | Papadimitriou | 2008 | 107 | 181 | 288 | 60 | 32 | 15 | 47 | 101 | 33 | 0.461 | 0.099 |  |
| 27 |  | Peplonska | 2017 | 188 | 451 | 639 | 74 | 82 | 32 | 162 | 222 | 67 | 0.395 | 0.521 |  |
| 28 |  | Roth Black | 2008 | 26 | 211 | 237 | 11 | 14 | 1 | 117 | 83 | 11 | 0.249 | 0.447 |  |
| 29 |  | Roth Western | 2008 | 52 | 668 | 720 | 13 | 34 | 5 | 218 | 317 | 133 | 0.436 | 0.362 |  |
| 30 |  | Ruiz | 2009 | 100 | 100 | 200 | 23 | 23 | 7 | 29 | 58 | 13 | 0.420 | 0.057 |  |
| 31 |  | Ruiz | 2011 | 66 | 334 | 400 | 14 | 43 | 9 | 104 | 169 | 61 | 0.436 | 0.596 |  |
| 32 |  | Ruiz | 2013 | 119 | 343 | 462 | 37 | 66 | 16 | 106 | 175 | 62 | 0.436 | 0.488 |  |
| 33 |  | Santiago | 2007 | 102 | 123 | 225 | 29 | 55 | 18 | 60 | 45 | 18 | 0.329 | 0.057 |  |
| 34 |  | Saunders | 2007 | 152 | 143 | 295 | 55 | 73 | 24 | 39 | 75 | 29 | 0.465 | 0.978 |  |
| 35 |  | Scott Jamaica | 2010 | 114 | 311 | 425 | 86 | 25 | 3 | 232 | 73 | 6 | 0.137 | 0.926 |  |
| 36 |  | Scott USA | 2010 | 113 | 190 | 303 | 79 | 32 | 2 | 126 | 57 | 7 | 0.187 | 0.861 |  |
| 37 |  | Sessa | 2011 | 29 | 45 | 74 | 11 | 11 | 7 | 17 | 21 | 7 | 0.389 | 0.903 |  |
| 38 |  | Wang Asian | 2013 | 166 | 1,252 | 1,418 | 57 | 79 | 30 | 323 | 640 | 289 | 0.486 | 0.413 |  |
| 39 |  | Wang Western | 2013 | 130 | 1,694 | 1,824 | 36 | 68 | 26 | 540 | 840 | 314 | 0.433 | 0.690 |  |
| 40 |  | Wessner | 2016 | 56 | 216 | 272 | 22 | 22 | 12 | 65 | 102 | 49 | 0.463 | 0.459 |  |
| 41 |  | Yang | 2003 | 107 | 436 | 543 | 53 | 48 | 6 | 130 | 226 | 80 | 0.443 | 0.292 |  |
| 42 |  | Yang | 2007 | 65 | 63 | 128 | 55 | 9 | 1 | 51 | 11 | 1 | 0.103 | 0.654 |  |
| 43 |  | Yang | 2017 | 59 | 50 | 109 | 29 | 27 | 3 | 13 | 20 | 17 | 0.490 | 0.168 |  |
| 44 |  | Yusof | 2016 | 41 | 180 | 221 | 19 | 17 | 5 | 40 | 103 | 37 | 0.492 | 0.052 |  |
|  |  |  |  |  |  |  |  |  |  |  |  |  |  |  |  |

🟋: Laplace-corrected genotype data; USA: United States of America; RR: common genotype; XX: variant genotype; RX: heterozygous genotype; maf: minor allele frequency; HWE: Hardy-Weinberg Equilibrium
