# Supplementary material for: Association of the ACTN3 R577X (rs1815739) polymorphism with elite power sports: A meta-analysis
Source: PLoS One. 2019 May 30;14(5):e0217390. doi: 10.1371/journal.pone.0217390 (PMC6542526; doi:10.1371/journal.pone.0217390)
Supplement: S5 Table — (DOCX) [file pone.0217390.s006.docx]

|  | I | Section name and paragraph number within manuscript |
| --- | --- | --- |
|  | **Introduction** |  |
| 1 | Provide a detailed justification for the polymorphism studied; if a single polymorphism was analyzed, give details as to why others were not included in the meta-analysis. | Pages 5-6, Lines 196-215 |
| 2 | Provide a detailed justification for the population(s) and clinical condition studied. | Page 6, Lines 196-215 |
|  | **Methods** |  |
| 3 | Provide full details of the search strategy employed; outline the full electronic search strategy –specific combination of keywords and any limits applied- for at least one database. Specify whether synonyms of polymorphisms/genes (e.g. SNP number) were searched. | Page 6, Lines 221-225 |
| 4 | Report full details on the inclusion and exclusion criteria applied for selecting studies.  *Please list the excluded articles and the reasons for exclusion of each article in a supplementary file.* | Page 6-7, Lines 225-234 |
| 5 | Provide details on how the quality of the studies included in the analyses was assessed. | Page 7-8, Lines 256-260 |
| 6 | Describe steps taken to contact study authors to identify additional studies and to request missing data. | Page 7, Line 246 |
| 7 | Describe how environmental effects were adjusted for, if this adjustment was not conducted, outline the reasons for this. | NA |
| 8 | Describe the methods of handling heterogeneity/between-study variance. | Page 9, Lines 289-295 |
| 9 | Describe how the Hardy-Weinberg equilibrium and linkage disequilibrium were assessed. | Page 7, Lines 251-252; S1 Table |
| 10 | Describe and justify the choice of model for the analyses (per-allele vs per-genotype vs genetic model-free, random effects vs fixed effects). | Page 8 , Lines 275-279 |
| 11 | Describe whether a sensitivity analysis has been completed. | Page 9, Lines 294-296 |
| 12 | Describe whether an assessment of the effects of population stratification has been conducted. | Page 9, Lines 289-  302 |
| 13 | Describe whether study-specific results have been assessed and if so the reasons for this (e.g. forest plot). | Page 8, Lines 279-287 |
|  | **Results** |  |
| 14 | Include flow diagram for the studies included in the meta-analysis as the first figure for the manuscript | Page 9, Lines 311-313; Fig 1 |
| 15 | Report details on allele/genotype prevalence. | Page 10, Lines 324-327; S3 Table |
| 16 | Report the effect size estimates and p values for each analysis. | Pages 11-15, Lines 354-440; Tables 1-4 |
|  | **Discussion** |  |
| 17 | Discuss the limitations of the meta-analysis, including genotyping errors/bias and publication bias. | Page 23, Lines 623-643 |
| 18 | If the meta-analysis identifies an association within a subgroup of the population studied but not another, discuss the implications of these results, and if applicable the possibility of subgroup-specific publication bias. | Pages 18-19, Lines 514-534 |
| 19 | Discuss the suitability of the sample size employed to the research question and the power of the study. | Page 17-18, Lines 486-493;  Page 23, Lines 636-637 |
