# Supplementary material for: Association of the ACTN3 R577X (rs1815739) polymorphism with elite power sports: A meta-analysis
Source: PLoS One. 2019 May 30;14(5):e0217390. doi: 10.1371/journal.pone.0217390 (PMC6542526; doi:10.1371/journal.pone.0217390)
Supplement: S6 Table — (DOCX) [file pone.0217390.s007.docx]

**S6 Table** **Comparisons among meta-analyses that examined *ACTN3* R577X polymorphism with power sports**

|  | **This study** | **Weyerstraβ [49]** | **Ma [10]** | **Alfred [13]** |
| --- | --- | --- | --- | --- |
| Year | 2018 | 2018 | 2013 | 2011 |
| Sports performance | Power | Power | Power/Endurance/Mixed | Power/Endurance/Mixed |
| Subgroups | Race/Gender | Race/Gender | Race/Gender | Race |
| Genetic modeling | Allele genotype | Recessive Dominant | Recessive Dominant | Recessive Dominant |
| **Methods, Treatments** |  |  |  |  |
| HWE as inclusion criterion | Yes | NM | No | No |
| Outlier analysis | Yes | No | No | No |
| Heterogeneity | Q, I^2^ | Q | Q, I^2^ | I^2^ |
| Interaction tests | Yes | No | No | No |
| Sensitivity analysis | Yes | No | No | No |
| Publication bias | Yes | NM | Yes | Yes |
| Other polymorphisms | No | Yes | Yes | No |
| **Significant overall findings** | | | | |
| Genetic comparison | R allele | R allele-based | RR versus RX+XX | RR versus RX+XX |
| Number of studies | PRO: 44 | 19 | 18 | 12 |
|  | PSO: 39 |  |  |  |
| OR | PRO: 1.21 | 0.79 | 1.21 | 1.41 |
|  | PSO: 1.20 |  |  |  |
| 95% CI | PRO: 1.07-1.37 | 0.69-0.91 | 1.03-1.42 | 1.23-1.62 |
|  | PSO: 1.12-1.30 |  |  |  |
| P-value | PRO: 0.002 |  |  |  |
|  | PSO: 10^-5^ |  |  |  |
| I^2^ (%) | PRO: 61 | NM | 44.82 | 73.5 |
|  | PSO: 23 |  |  |  |

HWE: Hardy-Weinberg Equilibrium; Q: Q test for association; OR: odds ratio; CI: confidence interval; PRO: pre-outlier; PSO: post-outlier; NM: no mention; RR: common genotype; XX: variant genotype; RX: heterozygous genotype

**References:**

10. Ma F, Yang Y, Li X, Zhou F, Gao C, Li M, et al. The association of sport performance with ACE and ACTN3 genetic polymorphisms: a systematic review and meta-analysis. PloS one. 2013;8(1):e54685. doi: 10.1371/journal.pone.0054685

13. Alfred T, Ben-Shlomo Y, Cooper R, Hardy R, Cooper C, Deary IJ, et al. ACTN3 genotype, athletic status, and life course physical capability: meta-analysis of the published literature and findings from nine studies. Human mutation. 2011;32(9):1008-18. doi: 10.1002/humu.21526

49. Weyerstraβ J, Stewart K, Wesselius A, Zeegers M. Nine genetic polymorphisms associated with power athlete status - A Meta-Analysis. Journal of science and medicine in sport. 2018;21(2):213-20. doi: 10.1016/j.jsams.2017.06.012
